# Indirect Enantioseparations: Recent Advances in Chiral Metabolomics for Biomedical Research

**DOI:** 10.3390/ijms23137428

**Published:** 2022-07-04

**Authors:** Luisa-Gabriela Bogos, Ioana-Ecaterina Pralea, Radu-Cristian Moldovan, Cristina-Adela Iuga

**Affiliations:** 1 Department of Proteomics and Metabolomics, Research Center for Advanced Medicine–MEDFUTURE, “Iuliu Hațieganu” University of Medicine and Pharmacy Cluj-Napoca, Louis Pasteur Street 6, 400349 Cluj-Napoca, Romania; bogos.luisa.gabriela@elearn.umfcluj.ro (L.-G.B.); pralea.ioana@umfcluj.ro (I.-E.P.); iugac@umfcluj.ro (C.-A.I.); 2 Department of Pharmaceutical Analysis, Faculty of Pharmacy, “Iuliu Hațieganu” University of Medicine and Pharmacy, Louis Pasteur Street 6, 400349 Cluj-Napoca, Romania

**Keywords:** chiral metabolomics, indirect chiral analysis, chiral derivatization agents, endogenous metabolites, mass spectrometry metabolomics, ion-mobility mass spectrometry

## Abstract

Chiral metabolomics is starting to become a well-defined research field, powered by the recent advances in separation techniques. This review aimed to cover the most relevant advances in indirect enantioseparations of endogenous metabolites that were published over the last 10 years, including improvements and development of new chiral derivatizing agents, along with advances in separation methodologies. Moreover, special emphasis is put on exciting advances in separation techniques combined with mass spectrometry, such as chiral discrimination by ion-mobility mass spectrometry together with untargeted strategies for profiling of chiral metabolites in complex matrices. These advances signify a leap in chiral metabolomics technologies that will surely offer a solid base to better understand the specific roles of enantiomeric metabolites in systems biology.

## 1. Introduction

Chirality is a key feature of biological systems, being an intrinsic property of biomolecules such as amino acids, sugars, and proteins. From a molecular perspective, chirality is defined by the presence of at least one chiral center (most frequently carbon or nitrogen atoms, with different substituents), resulting in isomers whose structures are not superposable called enantiomers.

In biological systems, chirality confers remarkably different activity due to distinct interactions of enantiomers in chiral environments. Several studies emphasized different chemical behavior of enantiomers relevant to pathologic conditions (e.g., neurological disorders, cancer, kidney diseases) [1,2], with enantioselective analyses nowadays being of considerable interest.

Ever since the discovery of D-Ser as an endogenous metabolite [3], D-amino acids (D-AAs) have been found to play significant roles in metabolism, with some of them reflecting the onset or progression of pathological states. Altered levels of D-amino acids have been reported in neurological and neurodegenerative diseases, such as schizophrenia [4], depression [5,6], Alzheimer’s disease (AD) [7], amyotrophic lateral sclerosis [8,9]. Being the most studied, D-Ser appears to have important neuromodulatory roles, as it is a more potent coagonist on the N-methyl-D-aspartate (NMDA) receptor than glycine. Considering D-Ser implications in NMDA receptor regulation, its use has been proposed and investigated in the treatment of schizophrenia and depression [10], as well as for treating anxiety disorders [11]. Additionally, D-Asp, usually found concentrated in the synaptic vesicles of terminal axon, had been identified as significantly decreased in the prefrontal cortex of patients with schizophrenia [4], while racemization to D-Asp, D-Ser, and D-Thr was associated with the development of cataracts [12].

Moreover, D-Ser has been recently highlighted as a promising biomarker for evaluating kidney functions [1,13,14,15]. In chronic kidney disease, D-Ser, D-Asn, and D-Pro levels are increased compared with healthy people, being strongly correlated with kidney function (glomerular filtration rate), while D-Asp and D-Pro may indicate the copresence of diabetes [16].

Implications of D-AAs in the development of several cancers have also been reported: high levels of D-Ala were reported in the gastric secretion of helicobacter pylori-positive gastric cancer patients [17]; a decrease in the levels of D-Glu and D-Gln were observed in patients with hepatocellular carcinoma [18]; while in vitro studies revealed significantly higher levels of D-Ser and D-Asp in MCF-7 breast cancer cells [19]. 

The incorporation of D-amino acids in peptides leads to dysfunctional proteins; such is the case of the amyloid-beta peptide toxic fragment associated with neurodegeneration in AD, which contains D-Ser at position 26; or the racemization of Asp58, Asp84, and Asp151 in α-crystallin, resulting in decreased solubility and function impairment and associated with the development of cataracts [20]. A more thorough discussion on the influence that D-Asp has on protein structure and function was recently published by Fujii et al. [21].

Besides amino acids, the second most popular research direction was the study of α-hydroxy acids’ metabolic roles. Salivary levels of D- and L-lactate (LA) have been proposed as diagnostic biomarkers for diabetes mellitus [22,23,24], which might prove to be a good matrix for population screening. Elevated levels have also been found in plasma of diabetic rats [25]. Both D- and L-enantiomers of 2-hydroxyglutarate (2HG) were observed to have implications in several forms of cancer, such as gliomas [26], breast cancer [27], myeloid leukemia [28], and some forms of brain cancer [29]. 

The analysis of chiral metabolites requires high sensitivity, in addition to good enantioselectivity, as the concentrations of D-AAs in biological samples (e.g., human serum) vary significantly, from trace concentrations of 0.025–0.1 µM for D-Arg, D-Glu, D-His, D-Met, D-Gln, D-Leu, and D-Phe; to more abundant ones of 0.11–0.9 µM (D-Asp, D-Asn, D-Ser, D-Val, D-Thr) and even higher for others of 0.9–5 µM (D-Ile, D-Ala) [18].

The direct approach to chiral metabolomics implies enantiospecific interactions between the analytes and a chiral selector. Separative techniques, including liquid chromatography (LC), gas chromatography (GC), and capillary electrophoresis (CE), that rely on the selectivity provided by chiral stationary phases and selectors have been extensively applied in the analysis of enantiomers [30,31]. Unfortunately, for most of these small polar molecules, it is difficult to achieve chiral resolution, and most importantly, considering their lack of fluorophores and chromophores, significant difficulties are encountered in their detection using optical techniques. Chiral derivatization can correct these issues by improving their molecular properties to be more suitable both for separation (which can be achieved in non-chiral environment) and for better detection sensitivity and specificity.

The main advantages presented by indirect enantioseparations include reduced cost, variable choice of detectors (ultraviolet (UV), fluorescence (FL), mass spectrometry (MS), and others), very good chiral selectivity, increased retention of the derivatives, more sensitive and selective detection, and facile control of derivatives’ elution order [32]. At the same time, significant drawbacks are represented by the increased time for sample preparation, as well as derivatization requiring a specific amount of time depending on the used chiral derivatizing agent (CDA). Moreover, if the CDA does not have high optical purity, additional stereoisomers might be formed and coelute with peaks of interest. Despite these, the indirect approach is the most widely used method for separation of chiral small molecules in biological samples [33].

The last comprehensive review regarding the indirect chiral analysis of proteinogenic amino acids and related metabolites was published in 2008 by Ilisz et al. [34]. Since then, this topic has been partially covered by other reviews, with some focusing on the chiral separation of amino acids [35,36,37], some on the role of some classes of analytes in certain diseases [33], and others describing the use of a specific CDA [32,38] or the enantioanalysis of metabolites by liquid chromatography [39].

In this review paper, we intend to provide a landscape of recent developments in the field of chiral analysis of endogenous metabolites using the indirect approach. Section 2 provides an overview of most popular CDAs, with a focus on newly developed labeling agents and their applications, while the third section presents new methodologies developed for untargeted profiling of chiral metabolites in complex matrices. Section 4 is focused on describing the state of the art in enantioseparations that target specific analytes or classes of analytes, with an emphasis on chromatographic and electrophoretic techniques. Recent progresses in ion-mobility mass spectrometry (IMS) separations of derivatized compounds are presented in Section 5.

## 2. Advances in Chiral Derivatization

**Figure 1:**
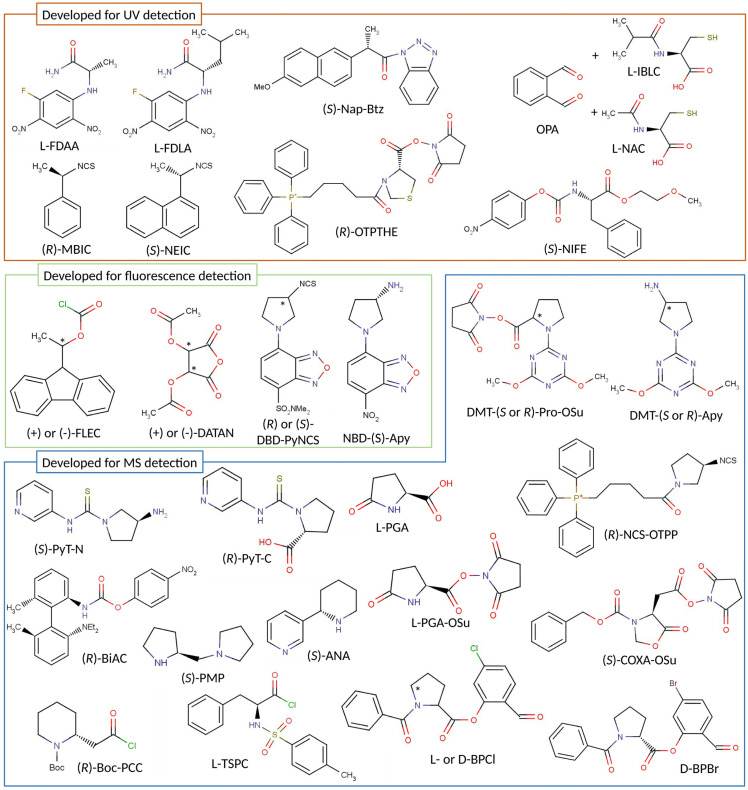
Chemical structures of reported CDAs grouped according to the detection method for which they were developed. CDAs presented as racemates can be used in both enantiomeric forms.

Most of the recently published studies employing the indirect approach were centered on chiral analysis of amino acids and α-hydroxy acids, with the research being focused on two main directions: improvements in the separation using existing CDAs and the development of new CDAs. Therefore, this section covers aspects of derivatization with both commercially available CDAs and newly synthesized ones. More relevant information regarding the structures, reaction conditions, and specific applications of CDAs can be found in Figure 1 and Table 1.

Considering that these chiral metabolites are polar and hydrophilic molecules, an ideal CDA should increase their lipophilicity for better retention on conventional reversed-phase stationary phases, while the derivatizing reaction should be fast and quantitative, without any racemization. CDAs should be available in both enantiomeric forms and be optically pure in order to avoid overestimation of trace chiral metabolites in complex matrices. Preferably, CDAs should also be commercially available or easy to synthesize.

Traditionally, derivatization agents were developed to improve UV or FL detection of certain metabolites, resulting in CDAs that have been successfully used for more than 30 years. However, some of these CDAs are starting to become obsolete, considering the increased availability of mass spectrometry and its advantages (high sensitivity and selectivity). Recently, efforts have been made toward developing new CDAs with better characteristics for MS detection (covered in Section 2.3), including better ionization properties or yielding intense daughter ions after MS/MS fragmentation.

Another relevant advance is represented by the implementation of isotopically labeled derivatization of CDAs for chiral metabolomics profiling. Their specific role in these approaches is described in Section 3.

### 2.1. Advances and Improvements in Chiral Derivatization

**Marfey’s reagent, or 1-fluoro-2,4-dinitrophenyl-5-L-alanineamide** (**FDAA**), has probably been the most successful CDA since its introduction in 1984 [40]. It reacts in alkaline conditions (NaHCO_3_; triethylamine (TEA)) with amino groups without racemization, yielding diastereomers. Details regarding FDAA synthesis and applications can be found in several reviews [41,42].

A new approach for the derivatization of α-hydroxy acids with FDAA was described by Moon et al. [43]. Considering that the nucleophilicity of the hydroxyl group is not strong enough, it must be increased in order for the reaction to take place. The method implied the addition of NaH (60% dispersed in oil) to a solution of α-hydroxy acids in tetrahydrofuran (THF) at room temperature. After 5 min, L-FDAA was added to this solution and stirred under argon or nitrogen. The reaction was quenched after 2 min using HCl, then analyzed using reversed-phase LC-MS.

In the reaction between FDAA and proteinogenic amino acids, kinetics can vary substantially among the analytes, with one explanation being the fact that many of them are doubly labeled. The reaction kinetics between FDAA and proteinogenic amino acids was assessed by Ayon et al. [44], who found that for quantitative analysis (yield > 95%), a 24 h incubation time is sufficient for all analytes except His (>78 h), while the CDA concentration should be at least 4 times higher than that of the analytes.

The hydrophobicity of the derivatives can be tuned by replacing the L-alanineamide moiety of FDAA with other amides of amino acids [45], such as L-leucylamide, resulting in 1-fluoro-2,4-dinitrophenyl-5-L-leucinamide (L-FDLA). Derivatization with this CDA was recently implemented in the analysis of D- and L-AAs in brain tissue samples [46], along with some improvements in derivatization [47] that consisted of using TEA instead of NaHCO_3_ to achieve the alkaline pH. In this way, TEA caused less ion suppression, resulting in improved detection sensitivity.

**Table 1:** Derivatization characteristics of most commonly reported CDAs.

CDA	Name	Derivatization Moiety	Derivatization Conditions	Commercially Available	Ref.
L-FDAA	1-Fluoro-2,4-dinitrophenyl-5-L-alanineamide	Amines	▪ Alkaline pH (TEA, NaHCO_3_), 24 h incubation for yield > 95%	Yes	[41,42]
		α-Hydroxy acids	▪ In presence of NaH (60% dispersed in oil); Sample in THF		
OPA	*o*-Phthalaldehyde/chiral thiols	Primary amines	▪ Alkaline pH (sodium tetraborate, NaOH) ▪ Chiral thiols: isobuteryl-L-cysteine or N-acetyl-L-cysteine	Yes	[48,49]
(+) or (−)-FLEC	(+) or (−)-1-(9-Fluorenyl)ethyl chloroformate	Primary and secondary amines	▪ Alkaline pH (sodium tetraborate) ▪ FLEC in acetone or ACN ▪ Excess reagent of at least 1:10 will ensure quantitative reaction	Yes	[38]
		Thiols			
(*S*)-NIFE	N-(4-nitrophenoxycarbonyl)-L-phenylalanine 2-methoxyethyl ester	Primary and secondary amines	▪ Alkaline pH (sodium tetraborate, TEA) ▪ CDA in acetone or ACN ▪ Excess reagent of at least 1:10 will ensure quantitative reaction	Yes	[50]
		Thiols			
		Phenols			
(*R/S*)-DBD-PyNCS	((*R*/*S*)-4-(3- isothiocyanatopyrrolidin-1-yl)-7-(N,N-dimethylaminosulfonyl)-2,1,3-benzoxadiazole	Primary and secondary amines	▪ In presence of TEA or DMAP, CDA dissolved in ACN	Yes	[32,51]
		Carboxylic acids	▪ In aprotic media, using condensation agents		
NBD-(*S*)-APy	(*S*)(+)-4-Nitro-7-(3-aminopyrrolidin-1-yl)-2,1,3-benzoxadiazole	Primary and secondary amines	▪ In presence of TEA or DMAP, CDA dissolved in ACN	No	[32,51]
		Carboxylic acids	▪ In aprotic media, using condensation agents		
(+)- or (−)-DATAN	(+) or (−)-Diacetyl-L-tartaric anhydride	Amines	▪ Reacts in aprotic media (CH_2_Cl_2_:acetic acid–4:1)	Yes	[52,53]
		Hydroxyls			
DMT-3(*S* or *R*)-Apy	(*S*)-1-(4,6-dimethoxy-1,3,5-triazin-2-yl)pyrrolidin-3-amine	Carboxylic acids	▪ In presence of activation reagents (TPP and DPDS)	No	[54]
DMT-1(*S* or *R*)-Apy	(*S*)-1-(4,6-dimethoxy-1,3,5-triazin-2-yl)pyrrolidin-1-amine	Carboxylic acids	▪ In presence of activation reagents (TPP and DPDS)	No	[54]
DMT-(*S* or *R*)-Pro-OSu)	((*S*)-2,5-dioxopyrrolidin-1-yl-1-(4,6-dimethoxy- 1,3,5-triazin-2-yl) pyrrolidine-2-carboxylate	Amines	▪ CDA in ACN, in presence of TEA ▪ Room temperature, 40 min	No	[55]
*(R*)-NCS-OTPP	(*R*)-(5-(3-isothiocyanatopyrrolidin-1-yl)-5-oxopentyl) triphenylphosphonium	Thiols	▪ In presence of TEA	No	[56,57]
(*S*)-COXA-OSu	(3-[(Benzoyloxy)carbonyl]-5-oxo-1,3-oxazolidin-4-yl)acetate	Amines	▪ In PBS (100 mM) prepared in ACN	No	[58]
L-PGA	L-Pyroglutamic acid	Primary and secondary amines	▪ In presence of activators (EDC/HOBt)	Yes	[59]
L-PGA-OSu	L-Pyroglutamic acid succinidimyl ester	Amines	▪ CDA in ACN with TEA; sample in ACN	Yes	[60]
(*R*)-BiAC	(*R*)-4-nitrophenyl N-[2′-(dimethylamino)-6,6′-dimehyl-[1,1′-biphenyl]-2-yl] carbamate	Amines	▪ In borate buffer (pH 8.8) diluted with ACN	No	[61]
*(R)*-OTPTHE	N-[1-oxo-5-(triphenylphosphonium)pentyl]-(*R*)-1,3-thiazolidinyl-4-N-hydroxysuccinimide ester bromide salt	Amines	▪ In ACN containing borate buffer ▪ 60 °C, 30 min	No	[62]
D-BPBr	1-Benzoyl-pyrrolidine-2-carboxylic acid 5-bromo-2-formyl-phenyl ester	Primary amines	▪ In H_2_O/ACN solution containing 0.05M PBS	No	[63,64]
L- and D-BPCl,	1-Benzoyl-pyrrolidine-2-carboxylic acid 5-chloro-2-formyl-phenyl ester	Amines	▪ In H_2_O/ACN solution containing 0.05 M PBS	No	[63,64]
(*R*)-Boc-PCC	(*R*)-1-Boc-2-piperidine carbonyl chloride	Amines	▪ In aqueous solution mixed with acetone.	No	[65]
L-TSPC	N-(p-toluenesulfonyl)-L-phenylalanine chloride	Amines	▪ In presence of TEA or Py	Yes	[66,67]
		Hydroxyl	▪ Selective derivatization of hydroxy in anhydrous ACN with Py; 25 °C, 10 min		
(*S*)-PMP	(*S*)(+)-1-(2-pyrrolidinylmethyl)-pyrrolidine	Carboxylic acids	▪ In presence of activation reagents (TPP and DPDS)	Yes	[23]
(*S*)-Nap-Btz	(*S*)-naproxen-benzotriazole	Amines	▪ CDA in ACN; sample in NaHCO_3_ ▪ In presence of TEA; microwave derivatization (45 s, 600 W)	No	[68]
(*R*)-MBIC	Benzyl-isothiocyanate	Amines	▪ Sample in NaHCO_3_	Yes	[69]
(*S*)-NEIC	Naphtyl-isothiocyanate	Amines	▪ Sample in NaHCO_3_; microwave derivatization (60 s, 600 W)	Yes	[69]
(*S*)-ANA	(*S*)-anabasine	Carboxylic acids	▪ In presence of condensation agents (DMT-MM)	Yes	[70]

Abbreviations: ACN, acetonitrile; CDA, chiral derivatization agent; DMAP, 4-N,N-dimethylaminopyridine; DMT-MM, 4-(4,6-dimethoxy-1,3,5-triazin-2-yl)-4-methylmorpholinum chloride; DPDS, 2,2′-dipyridyl disulfide; EDC, 1-(3-dimethylaminopropyl)-3-ethylcarbodiimide; HOBt, 1-hydroxy-1H-benzotriazole; PBS, phosphate-buffered saline; Py, pyridine; TEA, triethylamine; THF, tetrahydrofuran; TPP, triphenylphosphine.

***o*-Phthalaldehyde (OPA)**, a well-known derivatizing reagent used mainly for fluorometric determinations, was used in several studies in combination with chiral thiols such as isobuteryl-L-cysteine (IBLC) [71] or N-acetyl-L-cysteine (NAC) [72,73]. The basic pH of the reaction was achieved by using either sodium tetraborate or sodium hydroxide, while OPA and chiral thiols were dissolved in organic solvents such as methanol (MeOH) or acetonitrile (ACN). OPA/chiral thiol derivatization targets primary amines, impeding the analysis of cysteine and proline. To overcome this problem, Yokoyama et al. [73] implemented a two-step labeling approach, with primary amines being derivatized with OPA/NAC, followed by derivatization of secondary amino acids with (+)-FLEC. Considering that both CDAs react in similar conditions, the only prerequisite was the basic pH, which was obtained with a saturated sodium borate solution.

The chiral analysis of 17 proteinogenic amino acids after OPA/IBLC derivatization was realized by Müller et al. [71] in complex biological matrices such as serum, plasma, urine, and cecal content. The derivatives were found to be stabile for 40 min; therefore, the analysis needed to be performed immediately after derivatization.

**(+) or (−)-1-(9-Fluorenyl)ethyl chloroformate (FLEC)** was first introduced more than 30 years ago [74] and proved to be one of the most versatile CDAs; the derivatizing conditions and several applications were covered in a comprehensive review [38]. In brief, FLEC reacts with primary amines, secondary amines, and thiols, with fast reaction kinetics. Excess reagent (at least 1:10 molar ratio) will ensure quantitative derivatization reactions.

Over the last decade, there were several relevant studies that employed FLEC derivatization for metabolomics studies, targeting the chiral analysis of amino acids by LC [73,75], capillary electrophoresis (CE) [76,77,78,79,80], or IMS [81].

Pre- and in-capillary FLEC labeling approaches have been reported for the enantioanalysis of proteinogenic amino acids in standard solutions [79], cerebrospinal fluid (CSF) samples [78], or artificial CSF (aCSF) [76,77,80]. A straightforward derivatization procedure for amino acid standards that consisted of mixing FLEC and AA solution (containing 5 mM of sodium tetraborate) was described by Prior et al. [79], with the reaction yield being estimated at 93–97%.

Derivatization of CSF samples was achieved [78] without needing any preliminary sample preparation. In brief, sodium tetraborate was added to the CSF sample for pH adjustment, followed by the addition of FLEC, resulting in a molar ratio of AA/FLEC between 1:50 and 1:100. The reaction yield was determined to be above 97% after 10 min of reaction time. The same derivatization procedure was also applied before ion-mobility separation in a study published by Pérez-Miguéz et al. [81].

The solid-phase extraction (SPE) of FLEC derivatives of five biologically relevant amino acids (Ser, Asn, Asp, Gln, and Glu) from aCSF was documented by Moldovan et al. [80] using a hydrophilic–lipophilic balance (HLB) SPE sorbent, with successful extraction being achieved using MeOH with 0.1% ammonia. The matrix effect was found to be marginal and the extraction efficiency was above 89%, with a derivatization efficiency above 80%.

Two other CE studies [76,77] reported the development of in-capillary labeling of amino acids with FLEC. This approach has the advantage of completely automatizing the derivatization procedure, with the separation capillary being used as a reaction chamber. After injection, the mixing of the sample and CDA plugs was achieved by applying a low voltage (0.1–0.2 kV) for a defined amount of time. Considering that the amino acids were negatively charged (pH 9.2, adjusted with sodium tetraborate), during the mixing step they would migrate toward the CDA plug, facilitating the reaction. The derivatization rates were similar or better when compared to precapillary derivatization.

The capability of (+) or (−)-FLEC to react quantitatively in-capillary can significantly decrease the sample preparation needed before analysis and offer a more reproducible workflow (fewer human errors). Still, it has some downsides, such as a slightly decreased sensitivity and selectivity, while the need for sample preparation techniques is not fully eliminated (i.e., for complex matrices).

**N-(4-nitrophenoxycarbonyl)-L-phenylalanine 2-methoxyethyl ester ((*S*)-NIFE)** is a CDA that reacts with primary and secondary amines, thiols, and phenols. The reaction will take place under basic conditions that can be provided by either TEA or sodium tetraborate. Compared to other commercially available CDAs (Marfey’s reagent, Sanger’s reagent, OPA/IBLC, 2,3,4,6-tetra-O-acetyl-β-D-glucopyranosyl isothiocyanate (GITC), 1-phenylethyl-isothiocyanate (AMBI), and 4-(3-isothiocyanatopyrrolidin-1-yl)-7-nitrobenzofurazan (NBD-PyNCS)), (*S*)-NIFE provided a significantly higher detection sensitivity [50], a result contradicted by Hess [82], who reported that the general order of sensitivity was GITC>(*S*)-NIFE≈L-FDAA>OPA-IBLC.

A landmark study was published by Visser et al. [50] describing the LC-MS/MS analysis of all proteinogenic amino acids in plasma, urine, and CSF samples. Improvements have been also made in terms of derivatization conditions, such as the use of sodium tetraborate for pH adjustment, representing reaction conditions that have been used in all studies employing (*S*)-NIFE derivatization ever since. While considering chiral purity to be of the utmost importance in analyzing trace quantities of chiral metabolites, a quality-control procedure employing enantiopure L-alanine was also proposed and implemented. The same procedure was later used by Tian et al. [83] for the characterization of D-AAs in milk samples.

Three studies based on (*S*)-NIFE derivatization were published by the group of Yan Cui for the analysis of catecholamines [84] and D-AAs in rat plasma [85] and rat brain, respectively [86].

Edman-type **chiral benzofuran-derived CDAs** ((*R/S*)-4-(3- isothiocyanatopyrrolidin-1-yl)-7-(N,N-dimethylaminosulfonyl)-2,1,3-benzoxadiazole (DBD-PyNCS) and (*S*)(+)-4-nitro-7-(3-aminopyrrolidin-1-yl)-2,1,3-benzoxadiazole (NBD-(*S*)-APy)) have been employed for labeling amino acids and related metabolites [87,88,89], or α-hydroxy acids (D- and L-lactic acid (LA)) [24], resulting in highly fluorescent reaction products. These derivatizing reagents are known to react with primary and secondary amines in the presence of triethylamine, forming fluorescent thiourea derivatives. The derivatization of carboxylic acids is also possible, but requires condensation agents. A book chapter written by Toyo’oka [32] covered all the important aspects of derivatization that employ these CDAs.

An analysis of D-Ser [87], L-Trp, and L-kynurenine (L-Kyn) [88] was conducted in human serum after protein precipitation; the resulting supernatant was derivatized by adding a solution of DBD-PyNCS and 4-N,N-dimethylaminopyridine dissolved in ACN. After 20 min at 55 °C, the reaction was terminated by adding a mixture of water and ACN (4:1) containing 0.1% acetic acid. Subsequent anion-exchange SPE was needed for targeted extraction of the analytes.

NBD-(*S*)-APy derivatization of D- and L-LA was reported [24] in a nonaqueous medium: the samples were either prepared in or extracted with ACN, whereas the CDAs and condensation reagents were dissolved in N,N-dimethylformamide. The NBD-(*S*)-APy reaction was quantitative after 60 min at 60 °C.

**(+) or (−)-Diacetyl-L-tartaric anhydride (DATAN)** is a recognized CDA employed in the derivatization of hydroxy acids and amino acids, and is mostly used for the chiral analysis of D- and L-LA and D- and L-2HG acid in different matrices. It reacts in aprotic media (CH_2_Cl_2_:acetic acid–4:1) with hydroxy and amino moieties, forming stable diastereomers. Quantitative reactions will occur in hermetically sealed containers at elevated temperatures (70–80 °C). Various reaction times have been reported, with the majority being 30–40 min, and some up to 2 h [90,91]. Most of the studies published recently [92,93,94,95] that reported the analysis of D- and L-2HG were based on the method described in 2004 by Struys et al. [52] with slight variations or adaptations to the available laboratory equipment. Nevertheless, a new LC-MS method using this CDA was proposed in 2016 by Poinsignon et al. [53] and validated for clinical applications.

Chiral-labeling DATAN was used by Scheijen et al. [25] in the analysis of D- and L-LA by UHPLC-MS/MS in plasma and urine samples, a method later adapted by Mason et al. [96] for the analysis of L-LA in CSF.

A very recent publication [91] described a new strategy for untargeted chiral metabolomics involving (+) and (−)-DATAN labeling of amino and hydroxy acids. Here, the reaction conditions were optimized in terms of reaction time and reagent concentration. Two hours of reaction time was found optimal for quantitative derivatization, while using DATAN at a concentration of 75 mg/mL.

The use of well-established CDAs for new applications provides some important advantages, such as a better understanding of reaction conditions and better predictability toward finding the optimal separation conditions. However, some of these CDAs, which were initially designed for UV or FL detection, are beginning to become obsolete now that mass spectrometry is becoming widely available.

### 2.2. New CDAs for UV or FL Detection

A novel CDA, N-[1-oxo-5-(triphenylphosphonium)pentyl]-(*R*)-1,3-thiazolidinyl-4-N-hydroxysuccinimide ester bromide salt (OTPTHE), was developed by Han et al. [62] and was designed specifically for separation and selective detection of D- and L-amino acids using reversed-phase LC. OTPTHE reacted with amines in mild conditions (in ACN containing borate buffer), with the reaction being complete after 30 min at 60 °C.

The group of Bhushan et al. synthesized several new CDAs [68,69,97] with applications for the enantioseparations of biologically relevant metabolites. Microwave-assisted derivatization (MAD) was implemented in all three approaches. Ten dichloro-(*S*)-triazine CDAs and six mono-(*S*)-triazines with L-amino acids and amides as chiral auxiliaries were synthesized and tested [97]; the researchers managed to separate the enantiomers of 13 proteinogenic amino acids. It was observed that, in general, the CDAs with amino acids as chiral auxiliaries offered better enantioresolution and were less retained. The MAD was assessed with the reaction conditions optimized depending on the CDA and analyte. Optimization limits were set for microwave irradiation at 75–90% power for 50–100 s at pH 8–10 (NaHCO_3_) and a molar ratio analyte:CDA between 1:1 and 1:5. MAD was also implemented in the reaction between proteinogenic amino acid enantiomers and (*S*)-naproxen-benzotriazole ((*S*)-Nap-Btz) [68], resulting in a fast derivatization (45 s) in the presence of TEA.

Benzyl- and naphthyl-isothiocyanate (MBIC and NEIC) CDAs have been used for the chiral analysis of selenomethionine (SeMet) [69], providing a simple and effective alternative of chiral labeling that also leads to good chromatographic separation of D- and L-SeMet derivatives.

### 2.3. New CDAs for MS Detection

The degree of selectivity and specificity offered by mass spectrometry gave rise to specific needs and opportunities in terms of CDA developments. Therefore, a new class of CDAs with improved properties for MS detection has begun to appear.

The synthesis of two new triazine-type CDAs specially developed for mass spectrometry detection was reported for the first time in 2015, targeting two important classes of endogenous chiral metabolites. (*S*)-1-(4,6-dimethoxy-1,3,5-triazin-2-yl)pyrrolidin-3-amine (DMT-3(*S*)-Apy) and (*S*)-1-(4,6-dimethoxy-1,3,5-triazin-2-yl)pyrrolidin-1-amine (DMT-1(*S*)-Apy) were developed for the analysis of carboxylic acids [54], while ((*S*)-2,5-dioxopyrrolidin-1-yl-1-(4,6-dimethoxy-1,3,5-triazin-2-yl) pyrrolidine-2-carboxylate (DMT-(*S*)-Pro-OSu)) can label biogenic amines [54]. An overview of these CDAs and some of their applications was presented in a book chapter by Toyo’oka [98]. Briefly, DMT-1(*S*)-Apy and DMT-3(*S*)-Apy react with carboxylic acids in the presence of activation reagents (triphenylphosphine and 2,2′-dipyridyl disulfide), with the reaction temperature also playing an important role (60 °C offers much higher peak areas than room temperature). The reactivity of the two CDAs was determined to be similar, with equal reaction rates and yields between the enantiomers. In the end, DMT-3(*S*)-Apy was considered to be more efficient, offering a higher sensitivity, better peak shape, and higher resolution values.

DMT-(*S*)-Pro-OSu synthesis and DL-amino acid labeling was reported by Mochizuki et al. [55]. This CDA can be used for the chiral analysis of amines and amino acids due to its capability to react with amines in mild conditions at room temperature. The reaction with all types of amino acids (neutral, acidic, basic and aromatic) was quantitative after 40 min of reaction time, regardless of temperature.

Since their introduction, derivatization with DMT-3(*S*)-Apy and DMT-(*S*)-Pro-OSu has been implemented in the analysis of peptides containing isomerized aspartic acid (L-α-Asp, L-β-Asp, D-α-Asp, and D-β-Asp) in crystallin samples [99], in the development of nontargeted metabolomics strategies [100,101] (discussed in Section 3, and for chiral discrimination using ion-mobility mass spectrometry [102].

Another mass spectrometry CDA was developed by Ma et al. [56,57] for derivatization of thiol compounds (DL-cysteine, DL-homocysteine, and glutathione). (*R*)-(5-(3-isothiocyanatopyrrolidin-1-yl)-5-oxopentyl) triphenylphosphonium (NCS-OTPP) derivatization was performed by mixing a 5 mM NCS–OTPP solution and 5% TEA, then incubating at 60 °C for one hour. The resulting diastereomer contained a permanent positive charge, which enabled good selectivity and high detection sensitivity.

Bromine- and chlorine-labeled probes (1-benzoyl-pyrrolidine-2-carboxylic acid 5-bromo-2-formyl-phenyl ester (D-BPBr) and 1-benzoyl-pyrrolidine-2-carboxylic acid 5-chloro-2-formyl-phenyl ester (D-BPCl, L-BPCl)) possessing stereodynamic chiral recognition characteristics have been recently synthesized [63,64] and used for profiling of amino-containing metabolites in human biofluids. Under optimized conditions, the derivatization reaction took place in a water–ACN solution containing 0.05 M phosphate-buffered saline (PBS). The reaction was promoted by ultrasonication at room temperature (for D-BPBr) or at 4 °C (for D-BPCl and L-BPCl) for 20 min, followed by overnight incubation at the same temperature to ensure a complete reaction. The reaction products were diastereomeric Schiff bases, which are D-enantiomers that offer a higher response in mass spectrometry. Among the two types of CDAs, D-BPCl and L-BPCl offer better sensitivity and selectivity than D-BPBr.

Two new pyridylthiourea CDAs have been proposed by Nagao et al. [103] for enantioanalysis of amines and carboxylic acids. The reaction required triphenylphosphine and 2,2′-dipyridyl disulfide and was complete in 1 h at 60 °C. The resulting diastereomers could be easily separated in RPLC with high resolutions (>3.5).

A series of prolylamidepyridines were evaluated as CDAs for the enantioseparation of carboxylic acids in saliva samples using LC-MS [104]. The reaction required 1-(3-dimethylaminopropyl)-3-ethylcarbodiimide and dimethylaminopyridine at 60 °C for 1 h. Considering that these CDAs were designed for MS detection, excellent sensitivity and separation were achieved.

The synthesis of succinimidyl (3-[(benzoyloxy)carbonyl]-5-oxo-1,3-oxazolidin-4-yl)acetate ((*S*)-COXA-OSu) was reported by Sakamoto et al. [58]; the substance was suitable for labeling amines. A requirement was considered for the existence of a ring structure with a restricted binding rotation close to the reaction point. The collision-induced dissociation (CID) fragmentation revealed highly specific fragment ions that contained amino acid structures, providing higher specificity than other CDAs (e.g., (*S*)-NIFE).

L-pyroglutamic acid (L-PGA) [59] is a CDA for labeling chiral primary and secondary amines, offering a high detection sensitivity through mass spectrometry. It is commercially available, present in different isotopic forms, and reacts under mild conditions, avoiding racemization. The reaction takes place in ACN in the presence of activation reagents such as 1-(3-dimethylaminopropyl)-3-ethylcarbodiimide and 1-hydroxy-1H-benzotriazole, and requires 60 min to react quantitatively. As a further development, a method for the analysis of chiral amino acids based on derivatization with L-PGA-OSu (the succinidimyl ester of L-PGA) was published by the same authors [60], with the reaction taking place in ACN with TEA, with complete derivatization after 10 min at 60 °C.

The synthesis of axially chiral reagents was recently described by Harada et al. [61]; the idea was derived from the chiral catalysts used for enantioselective organic reactions. (*R*)-4-nitrophenyl N-[2′-(dimethylamino)-6,6′-dimehyl-[1,1′-biphenyl]-2-yl] carbamate ((*R*)-BiAC (biaryl axially chiral tag)) was able to offer a baseline resolution for all proteinogenic amino acids due to the effective chiral environment provided by the axially chiral biphenyl moiety. (*R*)-BiAC reacted with chiral amines in borate buffer (pH 8.8) diluted with ACN at 55 °C for 10 min. Reaction quenching was performed by acidifying the reaction mixture with formic acid. Enhanced sensitivity in the attomole range was offered by the presence of a urea bond that could be easily cleaved by CID and a dialkyl amino group that could be easily protonated by ESI. Later, an LC-MS method for analysis of (*R*)-BiAC-derivatized amino acids in urine was also established [105].

Targeted analysis of D-Ser was described by Xie et al. [65] after precolumn derivatization with (*R*)-1-Boc-2-piperidine carbonyl chloride. The reaction occurred at room temperature under stirring (1000 rpm) for 2 h, then another hour of incubation was needed after addition of trifluoroacetic acid. This approach offered a limit of quantification of 0.19 µM, adequate for D-Ser analysis in human plasma.

The acyl group in N-(p-toluenesulfonyl)-L-phenylalanine chloride (TSPC) will react with amines, alcohols, and carboxyl groups, thus the derivatization was implemented in the analysis of D- and L-2HG [66,67]. For the selective derivatization of alcohol moieties in α-hydroxy acids by TSPC, Cheng et al. [66] used anhydrous ACN as the reaction solvent and pyridine to neutralize the hydrochloric acid produced. It was observed that a complete reaction could be achieved in 5 min at 25 °C with a concentration of TSPC of at least 1.25 mM. TSPC-labeled L-2HG acid was stable for at least 12 h. Another derivatization optimization was performed by Zheng et al. [67], who concluded that the reagent solution should be 12 mM TSPC containing 5% pyridine, while an incubation time of 20 min at 25 °C should provide robust derivatization.

Enantiomers of LA and 3-hydroxybutyric acid (3HB) in saliva samples were analyzed by LC-MS after derivatization with (*S*)(+)-1-(2-pyrrolidinylmethyl)-pyrrolidine ((*S*)-PMP) [23]. The CDA and analytes reacted at room temperature in the presence of 2,2-dipyridyl disulfide and triphenylphosphine as activators, and required at least 90 min to be quantitative. The formed derivatives were highly responsive in ESI-MS and produced characteristic product ions that enabled sensitive detection. A study regarding the chiral separation of DL-2-HB and DL-3-HB was published by Cheng et al. [106]. In this case, the derivatization was performed at 60 °C for 90 min with an (*S*)-PMP concentration of 0.2 mM.

(*S*)-anabasine ((*S*)-ANA) was used as a CDA for analyzing chiral carboxylic acids [70], greatly improving the detectability of those compounds using MS (20–160-fold). The reaction required condensation agents such as 4-(4,6-dimethoxy-1,3,5-triazin-2-yl)-4-methylmorpholinum chloride, and was quantitative in 5 min at room temperature.

Zhang et al. [107,108] used N-tert-butoxycarbonyl-O-benzyl-L-serine (BBS) as a CDA for their ion-mobility studies of amino acids. BBS was chosen due to its particular structure, as it contains polarizable heteroatoms and bulky substituents close to the chiral center, which can facilitate the separation. Chiral recognition by IMS was achieved for enantiomers of tryptophan, phenylalanine, cysteine, and proline.

Considering that not many of the CDAs that are commercially available are perfectly suitable for MS detection, this new generation of CDAs designed for improved MS detection may pave the way toward better chiral metabolomics.

## 3. Untargeted Methodologies for Chiral Metabolomics

Several methodologies for profiling of chiral metabolites have been reported in the literature (Tables S1 and S2). Some of them include the use of isotope-labeled CDAs, allowing for fold-change determination of the diastereomers formed for each chiral metabolite in two distinct sample groups (e.g., disease vs. control). Basically, one of the sample groups is derivatized with a chiral derivatizing reagent (light CDA), and the other group with the isotope-labeled form of the chiral reagent (heavy CDA). A pool of the two group samples is analyzed using LC-MS/MS with either an untargeted (DDA mode, HR-MS) or targeted (triple quadrupole) approach. By using nonchiral stationary phases, both heavy and light variant of the diastereomers coelute; nevertheless, based on the mass difference, they can be differentiated by MS. Toyo’oka’s group has published extensively on this subject. In 2013, the group used L-PGA as a CDA for the enantioseparation of primary and secondary amine metabolites (with enantiomers of 1-phenylethylamine (PEA) being used as prototype molecules) [59] and L-PGA-OSu [60] for amino acid enantiomers. The separation efficiency was evaluated on a reversed-phase C18 stationary phase, and the diastereomers were analyzed by a triple quadrupole mass spectrometer. All diastereomers obtained after L-PGA derivatization achieved good chromatographic separation within 10 min and with resolution values between 1.6 and 6.8. However, the separations of the hydrophilic amino acids after L-PGA-OSu labeling were insufficient due to the weak retention in the stationary phase used. Nevertheless, for nine aliphatic and aromatic amino acids, good enantioseparation was achieved, with resolutions ranging from 1.95 to 8.05. Moreover, the isotope labeling strategy using light and heavy (isotope-labeled) CDA was implemented for the analysis of the target molecules spiked at different R/S ratios in rat [59] and human plasma, respectively [60], allowing for determination of the fold change in the metabolite enantiomers in two distinct sample groups. A few isotope-labeled CDAs have also been reported. Zhang et al. [109] used a stable isotope N-phosphoryl amino acid labeling strategy (named SIPAL) for quantitative profiling of amine-metabolites by LC-MS. Two isotopic CDAs were synthesized (^16^O_2_- (light) and ^18^O_2_- (heavy) N-diisopropyl phosphoryl L-alanine N-hydroxysuccinimide esters (_16_O/_18_O-DIPP-L-Ala-NHS)) and used for this purpose. The method reported was applied for the amine-containing metabolites in human urine, providing both identification and absolute quantification of the targeted class.

The approaches described above were applied mostly to known chiral molecules determined using untargeted data sets. Subsequently, in 2015, the group of Toyo’oka reported an LC-MS/MS-based separation of chiral metabolites using two different CDAs (each in two enantiomeric forms) [100]. Briefly, the method consisted of two steps [98]: (i) chiral metabolomics fingerprinting: labeling of amine- and carboxyl-containing metabolites with an enantiopure derivatization agent (DMT-(*S*)-Pro-OSu and DMT-3(*S*)-Apy, respectively) and determination of the diastereomers formed with a precursor ion scan using LC-MS/MS. In this first step, DMT-tag metabolites (no annotations, no distinction between chiral and nonchiral metabolites) with significantly altered profiles between groups were identified; and (ii) selective extraction of the chiral molecules: samples were pooled and divided into two groups, one labeled with R-CDA and the other with the CDA’s antipode. Chromatographically, achiral metabolites will elute as single peaks, while chiral ones will present two peaks with opposite elution order in the sample aliquots derivatized with opposite CDAs. The approach was applied successfully in the chiral metabolomics fingerprinting and extraction of carboxyl and amines in the brain homogenate of AD patients and diabetic saliva, identifying L-Phe, L-LA, and D-LA as possible biomarkers.

**Figure 2:**
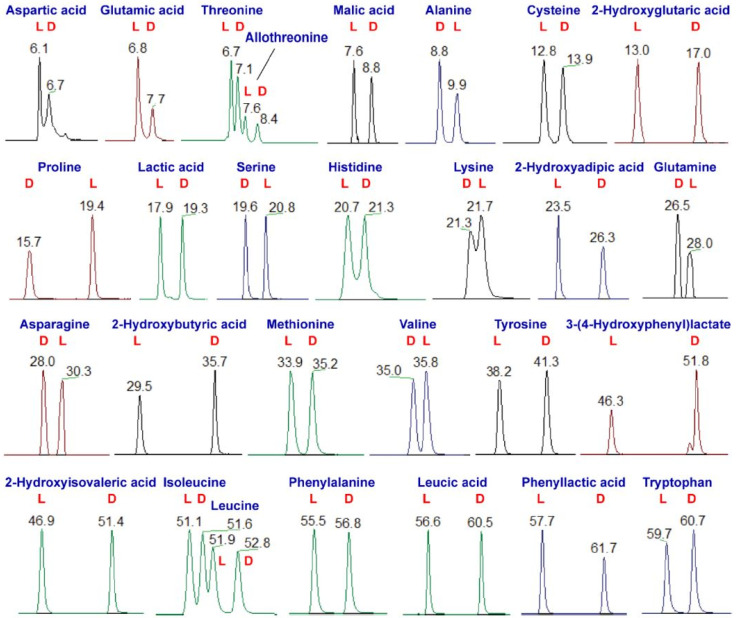
Base peak chromatogram showing baseline resolution of D- and L-enantiomeric forms of AAs and HAs using BEH C18 column. Reprinted with permission from Pandey et al. [91]. Copyright 2021, American Chemical Association.

Based on a similar workflow, DATAN was used for simultaneous derivatization of metabolites containing amine and hydroxyl groups [91], followed by separation and detection using reversed-phase LC-MS (Figure 2). Differentiation between achiral and chiral metabolites was made using MS/MS fragmentation patterns and the reversal of the elution order of metabolites labeled with the +/− enantiomeric forms of the CDA. The method was used for chiral metabolic profiling of acute myeloid leukemia (AML) patients, and was applied on peripheral blood and bone marrow plasma samples. A total of 67 chiral metabolites (D enantiomer of 22 amino acids and 8 hydroxy acids) were detected in the bone marrow plasma, while 65 chiral metabolites were identified in peripheral blood plasma (D enantiomer of 20 amino acids and 6 hydroxy acids were detected). Notably high levels of several D-amino acids and hydroxy acids characterized these samples. Moreover, the authors investigated the effect of chemotherapy on the metabolic response of the AML patients.

In order to provide a more accurate quantitative determination of isomer ratios available in trace amounts, Takayama et al. [101] employed the ^13^C_2_- isotope-coded derivatization agents (iCDAs) iDMT-(*S*)-Apy and iDMT-(*S*)-Pro-OSu, in addition to DMT-(*S*)-Apy and DMT-(*S*)-Pro-OSu. Furthermore, the group coupled this strategy with a data-dependent MS/MS analysis (DDA) acquisition mode derived from Q-TOF MS, allowing for simultaneous analysis of amines and carboxylic acids. Briefly, a pool prepared from all samples was derivatized using heavy iCDAs corresponding to the target class (iDMT-(*S*)-A for carboxylic acids or iDMT-(*S*)-PO for amines) and mixed in equal volumes, with each study sample labeled with the light versions of the CDAs. The authors used MSDIAL software for peak picking, deconvolution, and alignment for full MS and MS/MS scanning, as well as an Excel macro program for discrimination of isotope-coded/noncoded peak pair analysis (DINA), allowing the selective extraction of CDA-characteristic product ions and calculus of peak-intensity ratios of the light and heavy derivatives for subsequent multivariate analyses. The performance of the method was first tested on human serum spiked with different ratios of D/L enantiomers (D/L ratios of 0/100, 0.5/100, 1/100, and 2/100) of several prototype molecules. The iCDA correction improved accuracy, allowing differentiation between spiked metabolites’ intensity profiles at D/L ratios of 0/100 and 0.5/100. The method was improved significantly by the Q-TOF platform, achieving a 2 to 400 times higher sensitivity than reported previously by the group using a triple quadrupole MS. The optimized method was applied to the cerebrospinal fluid of AD patients for biomarker discovery, identifying 402 carboxylic-acid- and 629 amine-containing metabolites. The PCA of the metabolome comparison between the groups (*AD, non-AD) permitted identification of 9 carboxylic acids and 15 amines as biomarker candidates, with 8 compounds possessing chiral properties. Based on the MS/MS spectra, 16 compounds were annotated, with 6 of them being chiral.

Although the focus is truly on coverage of certain chiral metabolite classes (e.g., amino acids and hydroxy acids), remarkable improvements in terms of matrix-effect correction, accurate measurement of the isomer ratios of chiral isomers present in trace amounts, and overall approaches to accurate determination of biomarkers for reliable chiral metabolite separation applicable to untargeted metabolome studies were reported.

## 4. Targeted Analysis of Chiral Metabolites

### 4.1. Approaches Applicable to Chiral Profiling of Proteinogenic Amino Acids

The role of certain D-AAs in systems biology has not been deciphered yet. Therefore, enantiospecific analysis methods that can provide a comprehensive overview over the proteinogenic amino acids are needed in order to form new hypotheses regarding their roles.

The most common separation technique implemented for the indirect enantioanalysis of metabolites was liquid chromatography, most often coupled with mass spectrometry. In most cases, C18 stationary phases were preferred for the separation of hydrophobic diastereomers, together with binary mobile phases, usually formed of ACN and/or MeOH and sometimes THF [24], together with a buffer solution compatible with the detector. However, in certain cases, the nature of the stationary phase was crucial to achieving the desired level of separation. For example, DMT-Pro-Osu derivatives of serine could be separated only on an adamantylethyl stationary phase [55] that provided a higher polar surface. Other studies reported phenyl-based stationary phases to be more suitable for the separation of (*R*)-BiAC [61] and (+)-FLEC [75] derivatives, considering the aromatic nature of the analytes. For more information, complete data on separation conditions for the studies included in this review are available in Supplementary Materials Table S1.

Several studies reported the simultaneous chiral analysis of all 19 diastereomer pairs of proteinogenic amino acids after labeling with (*S*)-NIFE [50], (*R*)-BiAC [61], OPA/FLEC, or (*S*)-COXA-OSu [58] in matrices such as plasma, urine, or cerebrospinal fluid. Other noteworthy approaches that described the simultaneous separation of at least 10 enantiomer pairs of proteinogenic amino acids employed CDAs such as DMT-Pro-OSu [55], (+)-FLEC [75,76,77,78,79,80], (*S*)-Nap-Btz [68], OPA/IBLC [71], D-BPBr [63], and D-BPCl [64]. Some of these approaches can be categorized as high throughput, due to the fact that they manage to combine a fast analysis speed with the generation of high-quality data.

An analysis method developed by Visser et al. [50] targeted all 19 enantiomer pairs of proteinogenic amino acids labeled with (S)-NIFE, obtaining resolutions higher than 2.45 in less than 30 min. The method was validated for human cerebrospinal fluid, plasma, and urine, and was later adapted by other groups for the chiral analysis of amino acids in different matrices, such as rat plasma [85], rat brain homogenates [86], or milk [83].

The enantioanalysis of more than 40 α-AAs after derivatization with (*S*)-COXA-OSu derivatives was reported by Sakamoto et al. [58]. The formed diastereomers were separated on a triazole-bonded column, which interacted selectively with carboxylic moieties. Due to its lipophilic nature, the (*S*)-COXA-OSu was eluted at the beginning of the chromatogram, avoiding possible interferences with the compounds of interest. [58].

**Figure 3:**
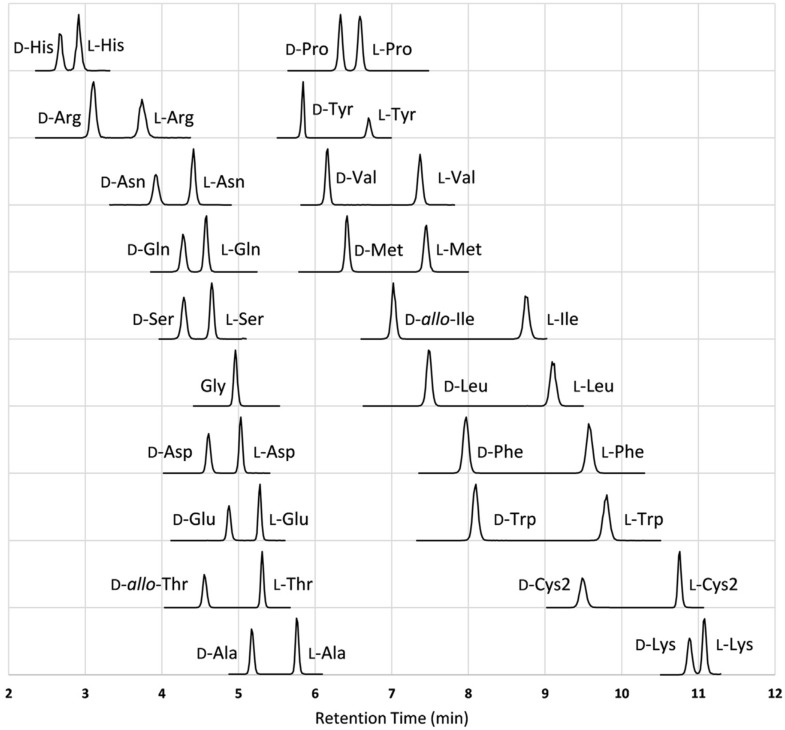
Peak separation of (*R*)-BiAC-tagged d- and l-amino acids under optimum separation conditions. Reprinted from Harada et al. [61], Copyright 2019, with permission from Elsevier.

Harada et al. [61] developed a new CDA, (R)-BiAC, and used it for the simultaneous enantioseparation of all 19 proteinogenic AAs with resolutions higher than 1.9 (except for *allo*-isomers) within 18 min on a phenyl stationary phase (Figure 3). The dialkyl amino group conferred an enhanced detection sensitivity to the diastereomers formed with this CDA, while good enantioselectivity was provided by the effective chiral environment created by the axially chiral biphenyl moiety. The same group adapted the LC-MS/MS method used previously for the analysis of 18 chiral proteinogenic AAs and Gly from human urine samples, achieving their separation in 20 min with a modified gradient and a longer column (75 mm instead of 50 mm). The method was validated using pooled human urine samples [105].

The use of phenyl-based stationary phases was also reported by Moldovan et al. [75]. The authors compared the performance of biphenyl and diphenyl stationary phases for the separation of proteinogenic AAs derivatized with (+)-FLEC. After optimization, baseline separation for 17 AAs was obtained on the biphenyl stationary phase within 30 min using ACN as an organic modifier. FLEC derivatization was used in several studies targeting the chiral analysis of amino acids using CE coupled with different types of detectors, such as UV [76], laser-induced fluorescence (LIF) [79], or MS [77,78,80]. This CDA proved to be very versatile, forming hydrophobic diastereomers and offering excellent enantioselectivity [38].

Fradi et al. [76] implemented a fully automated micellar electrokinetic chromatography (MEKC) approach with in-capillary derivatization of amino acids with (−)-FLEC, followed by the separation of the derivatives using a background electrolyte (BGE) formed with sodium tetraborate buffer, sodium dodecyl sulphate, and 2-propanol. Chiral resolution for 14 proteinogenic amino acids was achieved in 80 min, with limits of detection in the low micromolar range. On the other hand, Prior et al. [79], used precapillary derivatization with (+)-FLEC and a similar BGE (with LIF detection) to achieve chiral separation of 12 proteinogenic amino acids within 40 min. The group reported lower limits of detection (in the nM range).

Two micellar electrokinetic chromatography methods coupled to MS detection [77,78] separated FLEC diastereomers by using an MS-friendly volatile surfactant (ammonium perfluorooctanoate), achieving chiral resolution for the D- and L-derivatives of 14 proteinogenic amino acids, with sensitivity in the low micromolar range. Moreover, the approach developed by Moldovan et al. [77] was fully automatized by performing in-capillary derivatization.

Good enantioresolution for 18 proteinogenic amino acids was reported by Bhushan et al. [68] using HPLC-UV after derivatization with (*S*)-Nap-Btz, with the CDA providing increased lipophilicity due to its naphthyl moiety. Another HPLC method, this time coupled with an FL detector, was reported by Yokohama et al. [73]; the group proposed a two-step labeling procedure: first, the primary amines were labeled using OPA/NAC, then the secondary amines were derivatized with FLEC. In this way, the separation of all proteinogenic AAs was achievable within 90 min on a reversed-phase stationary phase.

OPA-based derivatization was performed also by Müller et al. [71], who developed and validated an LC-MS method using OPA/IBLC for precolumn derivatization for proteinogenic DL-AAs analyzed from complex matrices (human serum, plasma, urine, and mouse gut). Baseline separation was achieved for almost all the AAs, with the exceptions being Asp derivatives, which were not retained; Cys derivatives, which could not be detected; and Pro enantiomers, which do not react with this CDA due to the secondary amine.

A recent study reported the use of a newly synthesized CDA, DMT-Pro-OSu, for indirect enantioseparations of AAs in biological samples [55]. This method was applied to saliva samples from healthy volunteers to simultaneously analyze all proteinogenic AAs, achieving resolutions between 0.8 and 9.0. This was the first attempt to develop a new method for simultaneous and enantioselective determination of AAs from saliva using LC-MS, presenting a high sensitivity and good specificity [55]. By applying the same approach, Mizuno et al. [99] determined the positions of isomerized Asp residues in α-crystallin protein extracted from bovine eye lens.

Two similar methods that employed new CDAs were developed for analysis of AAs and amino-containing metabolites based on derivatization with two novel chiral bromine- and chlorine-containing aldehyde probes named D-BPBr [63] and D-BPCl [64]. These reagents showed preference for D-AAs, based on the derivatization efficiency between D- and L- forms and the MS response for the two corresponding peaks. Moreover, better chromatographic resolution was achieved for AAs with nonpolar (Ile, Val) and aromatic (Trp, Phe) substituents. Therefore, 14 pairs of AA enantiomers were separated and quantified, with D-BPCl offering a slightly better sensitivity and chiral selectivity. Both methods were validated for biological samples and proved useful in the analysis of trace levels of D-AAs in complex matrices such as urine and plasma.

In the case of DBD-PyNCS-DL-AA derivatives, two gradient elution systems were implemented in order to achieve chiral resolution for 17 DL-AA in nail samples, with resolutions between 1.62–6.96 [110]. It was concluded that DBD-PyNCS offered a higher resolution for neutral and aromatic AAs than for basic and acidic ones. In addition, for separation of the hydrophilic AAs, an isocratic elution with water and 30% MeOH in ACN containing trifluoroacetic acid seemed to offer a better resolution [32].

Even though many studies targeted the indirect analysis of the entire class of AAs, not many managed in the end to provide a baseline separation for all proteinogenic AAs. Nevertheless, the usefulness of these approaches is undoubted, and precious information regarding D-AAs has been reported.

### 4.2. Targeted Analysis Methods for Amino Acids

Generally, targeted methods aim at validating a certain hypothesis. This was also the case for indirect chiral separation methods, which aim to quantify D- and L-amino acids. Several methods have been developed for the quantification of various D-AAs in different samples, such as D-Ser in plasma [62,65,87,111], DL-Cys and DL-homocysteine (DL-Hcy) [56,57], L-Trp and L-Kyn in serum [88,89], or some amino acids relevant to neurometabolomics, such as DL-Ser, DL-Asp, DL-Asn, DL-Glu, and DL-Gln [80].

Xie et al. [65] developed a targeted approach for D-Ser analysis in human plasma after derivatization with (*R*)-1-Boc-2-piperidine carbonyl chloride. The low detection limit (LLOQ of 0.19 µM) and increased specificity provided by MS/MS detection made this approach suitable for quantification of D-Ser in plasma. The method was later applied by McGarry et al. [111] to plasma and CSF samples of Huntington’s disease patients.

An LC-MS/MS method validated for the analysis of (*S*)-DBD-PyNCS derivatives of D-Ser in human serum was described by Sakamoto et al. [87]. In this case, a triazole-bonded silica-packed column was used because this type of stationary phase offers good separation for acidic compounds due to the hydrophilic and anion-exchange interactions with protonated triazole.

A recently published study [62] that employed OTPTHE derivatization of AAs reported the successful separation of 13 pairs of AA diastereomers using RP-HPLC-UV, recording resolutions between 1.6 and 2.5 with a run time of 30 min. However, this method was only validated for the analysis of D- and L-Ser in human plasma.

Thiol compounds related to different diseases were enantioseparated after derivatization with (*R*)-(5-(3-isothiocyanatopyrrolidin-1-yl)-5-oxopentyl) triphenylphosphonium (NCS-OTPP) [56,57]. Chiral resolution of DL-Cys and DL-Hcy were achieved on a high-density C18 hybrid silica stationary phase, which offered better retention characteristics than other C18 columns. Using an isocratic elution, the separation was achieved in 25 min.

A capillary zone electrophoresis–mass spectrometry (CZE-MS) method was developed by Moldovan et al. [80] for the analysis of D- and L-enantiomers of five biologically relevant chiral amino acids: Ser, Asp, Asn, Glu, and Gln, after derivatization with (+)-FLEC. Separation was found to be highly dependent on the pH of the BGE, demonstrating that chiral discrimination was promoted by different pK_a_ values of the ionizable moieties of the diastereomers. Baseline separation was achieved for all five pairs of diastereomers, with sub-micromolar sensitivity.

(*S*)-DBD-PyNCS was used for the derivatization of L-Trp and L-Kyn [88,89] in application to human serum samples. L-Trp and L-Kyn were determined with resolution values of 2.22 and 2.13, respectively, and excellent detection sensitivity by FL (in nM range) [89]. This method was useful for evaluating the in vivo activity of indoleamine 2,3-dioxygenase or tryptophan 2,3-dioxygenase, important in patients with psychiatric disorders [88]. Zhou et al. used an LC method with chemiluminescence (CL) detection for the separation of Trp enantiomers, obtaining complete separation and low detection limits, but with a long analysis time [72].

### 4.3. Targeted Analysis Methods for α-hydroxy Acids

α-Hydroxy acids are the second most important class of chiral metabolites, and have been recognized to play significant roles in biological systems; in recent years, their analysis was often reported in human biological samples.

Several studies developed new approaches for the analysis of D- and L-LA in human saliva, since it was found to be a diagnostic biomarker in diabetic patients [23]. Therefore, D- and L-LA have been enantioseparated after derivatization with (*S*)-PMP [23], DBD-(S)-APy [22], NBD-(*S*)-Apy [24], or newly synthesized DMT-3(*S*)-Apy [54], PCP2 [104], PyT-C, and PyT-N [103]. All of these studies provided adequate separation of the analytes, with the most noteworthy being the study published by Tsutsui et al. [23], in which the recorded enantioresolution was 4.9, while the analysis time was only 7 min.

A cost-effective alternative for diabetes mellitus screening was developed by Numako et al. [22,24] for the analysis of LA enantiomers in saliva samples collected from dried saliva spots. The group reported two methods employing different CDAs, DBD-(*S*)-APy and NBD-(*S*)-Apy, with the latter offering higher chiral selectivity.

In addition to DL-LA, a few other chiral hydroxy acids were also analyzed, such as DL-2HB [106], DL-3HB [23,54], and malic acid [104].

Higashi et al. [70] used (*S*)-ANA as a CDA for the derivatization of chiral carboxylic acids in neonatal dried blood and saliva ((3-hydroxypalmitic acid and 2-(β-carboxyethyl)-6-hydroxy-2,7,8-trimethylchroman). It was reported that the CDA enhanced the detectability of the analytes by 1–2 orders of magnitude, with good enantiomeric separation (R_s_ of 1.92 and 1.75, respectively).

Enantiomers of 2HG were shown to be involved in diseases such as renal cancer [93] or AML [53,112]. A method using L-DATAN labeling offered baseline separation for these analytes in human serum and plasma [53]. TSPC was also used for labeling 2HG enantiomers, offering an improved chromatographic separation and detection sensitivity compared to L-DATAN [66,67]. The method was validated and then applied in the analysis of D- and L-2HG in human urine and tissue samples [66], as well as in rat serum and synovium tissue [67].

Several applications combined the analysis of α-hydroxy acids and amino acids, either derivatized with the same CDA (FDAA, for the determination of absolute configuration of a natural depsipeptide [43]) or with CDAs specific to each metabolite class, such as the one applied in the detection of chiral amino and α-hydroxy acids in brain homogenates from patients with Alzheimer’s disease [100].

## 5. Diastereomers Discrimination by Ion Mobility

Ion mobility coupled with mass spectrometry is a promising tool for gas-phase enantiomeric analysis that may address the limitations in chiral selectivity of previously described techniques. Briefly, in IMS, gas phase ions are separated based on their mobility as they are directed through a buffer gas under the influence of an electric field [113]. The mobility of an ion varies according to its mass, charge, and collision cross section (CCS), the latter being related to the ion’s size and conformation in the gas phase. Accordingly, coupling IM with MS provides an additional dimension to the separation, allowing ions with different CCS values to be separated even if they are isobaric or isomeric [114].

Several IMS technologies have been used for chiral analysis of small molecules that can be classified by means of separation as following: temporary dispersive separation techniques (drift-tube ion-mobility spectrometry (DTIMS), traveling-wave ion-mobility spectrometry (TWIMS), trapping with selective release (trapped ion-mobility spectrometry (TIMS), and spatially dispersive (field-asymmetric ion-mobility spectrometry (FAIMS)) [115]. Concisely, in DT-IMS, ions organized in packs are directed through a stationary buffer gas under low and constant electric field conditions [114]. In these terms, ions are separated based on their velocity in the carrier gas; in other words, ions with compact structures have fewer interactions with the drift gas, consequently traveling through the drift tube faster than larger structures would. Additionally, in TWIMS, a DC voltage pulse is applied in a series of sequentially and opposite polarity RF-only rings, creating traveling waves that separate ions based on their mobility in the gas-filled cell. [114,116,117]. Due to the reverse gas flow, the low-mobility ions are overtaken more often, hence increasing their transit times, while ions with high mobilities are “surfing” with the wave.

TIMS is a technique that traps ions against a moving gas using radially confining RF voltages and an axial electric field [118]; ions are then selectively ejected as the axial electric field is progressively decreased [115]. While the ion-mobility separation takes place in the second part of the TIMS tunnel, new ions are collected in the first section.

In contrast to DTIMS and TWIMS, FAIMS (differential mobility spectrometry (DMS) and differential mobility analyzer (DMA)) operates using high electric fields, making ion mobility dependent on the strength of the applied electric field; in fact, this dependence is measured, rather than the absolute value of the mobility. Accordingly, FAIMS uses a sequence of strong and weak electric fields to separate the gas-phase ions carried by a flow of buffer gas. Spatial dispersion is created by alternating high and low electric fields; therefore, an ion will oscillate between traveling to one electrode or another as the field fluctuates in polarity [113,119]. Only ions with a stable flight path will exit the FAIMS interface. A secondary DC voltage (termed the current compensation voltage (CV)) applied to FAIMS electrode in order to compensate for ion drift under varying field conditions will allow specific ion groups to traverse the interface for MS analysis [120].

The separation efficiency of these different IM techniques can be compared by defining the IM’s resolving power (Rp), usually reported in terms of CCS values and particularly, in FAIMS, in terms of compensation voltage. Accordingly, a direct comparison of resolving power values between FAIMS and IMS technologies operating in low electric fields is not suitable [115], since the separation parameters differ. Otherwise, the IMS Rp (expressed as CCS/ΔCCS) ranges from 40 to 50 in DTIMS and TWIMS, to 200 in TIMS, and even 750 in cyclic IMS [115], allowing separations of isomers and conformers with a 0.5% difference in CCS or higher [121].

Various IMS strategies were implemented in the analysis of enantiomers: (i) use of a chiral gas modifier to promote in situ formation of diastereomeric clusters with the enantiomer’s ions; (ii) use of an enantiopure amino acid as a chiral selector complexed with a divalent metal ion, forming trimers with the enantiomer [122]; (iii) use of a modified amino acid as a chiral selector to form metal-free dimers with the enantiomers [108]; and (iv) use of CDAs for diastereomer formation. Herein, we focused on the indirect IMS methods applied in the chiral analysis of small molecules (Table S3). A selection of the most recent advances in applying IMS separation for the chiral analysis of endogenous metabolites is discussed hereafter.

### 5.1. Enantiopure AAs Complexed with Divalent Metal Ions as Chiral Selectors

Several enantiopure amino acids as chiral selectors complexed with divalent metal ions were tested for the FAIMS-MS chiral separation of six amino acids [123]. First, optimization of the FAIMS parameters known to affect the resolving power (carrier gas composition -%He, position of the inner electrode, and makeup gas flow responsible for the gas flow rate through the interface) was carried out using [Ni^II^(refL-Asn)_2_(L-Trp)-H]^+^ and [Ni^II^(refL-Asn)_2_(D-Trp)-H]^+^ as prototypes. Several observations were made based on the optimization performed in this study: (i) the peak resolution tended to increase with an increase in the percentage of He in the carrier gas; (ii) the resolution might be improved with increasing tip distance, although ion transmission might be affected; and (iii) a low flow of makeup gas resulted in a slightly superior resolution, while ion transmission was severely affected at high flows. The paper [123] also presented the results of FAIMS separations of six DL-amino acids. Nine reference compounds along with four metals were screened for their ability to form [M^II^(refL-AA)_2_(^D/L^AA)-H]+ complexes separable in FAIMS. The tested aromatic amino acids (Trp and Phe) were more easily separated; however, their performance as reference compounds was unexceptional. L-Gln and L-Pro used as reference compounds attained successful separations of aromatic, neutral nonpolar, and basic amino acids included in the study (Trp, Phe, and Pro and Arg, respectively) while copper appeared to be the most successful metal (compared to Ni, Mg, and Zn).

In another study, Domalain et al. [122] used TWIMS for the differentiation of the D- and L-enantiomers of aromatic amino acids through cationization with copper (II) and multimer formation with D-proline (used as a chiral reference compound) in the form of [^D/L^AA + (D-Pro)_2_ + Cu^II^ − H]^+^ ions. In terms of the chiral reference compound, 11 amino acids were tested, while Phe was used as the prototype aromatic amino acid. ^D/L^Phe/Y/Cu^II^ ratio was optimized to obtain heterodimers and trimers, and the experimental CCS were recorded. D-Pro provided the highest CCS difference between the Phe’s D- and L-enantiomers, both in the heterodimer (ΔCCS 1.4 Å^2^) and trimer forms (ΔCCS 3.8 Å^2^), while no adducts were formed by the use of Ala, Gln, Cys, or Lys as chiral reference compounds. Independent of the chiral reference amino acid used, the highest Δt_d_ and ΔCCS were observed for the heterotrimers. In this sense, the authors obtained a good correlation between the D/L ratio of the respective [(^D^Pro)2 + ^D/L^Phe + Cu^II^ − H]^+^ ions and the mixture drift time. A very interesting approach was further implemented: FMOC was used to test if an improvement in enantiomer differentiation could be attained. Indeed, the analysis of the heterotrimers obtained after FMOC derivatization of Phe ([(^D^Pro)2 + ^D/L^Fmoc-Phe + Cu^II^ − H]^+^) slightly increased the Δt_d_ (from 0.11 to 0.16 ms), but this was not sufficient to increase the enantiomer differentiation. D-Pro was tested in the case of enantiomer separation of other nine amino acids, namely Arg, Trp, Glu, Thr, Gln, Tyr, Lys, His, and Cys. The highest difference in both drift time and CCS was observed in the case of the heterotrimers of the aromatic amino acids DL-Trp and DL-Tyr, while no adducts were formed with His and Cys.

Yu and Yao [124] also reported the use of TWIMS, this time to separate amino acid tetramers formed by complexation with binuclear copper and several L-enantiomeric amino acids as chiral selectors in the form of [(Cu^2+^)_2_XY_3_ − 4H + H/Na]^+^. Significant chiral discriminations were reported when using L-His as the chiral selector for Trp, Gln, Tyr, and Thr, achieving peak-to-peak resolutions (Rp-p) > 0.7. Instead, for His as the analyte, using Phe as the chiral selector was able to obtain an Rp-p of 0.693. Trp performed well as a chiral selector in the chiral discrimination of Gln, Glu, Met, Phe, and Tyr. The chiral selector Tyr discriminated between enantiomers of the basic amino acids Arg and His and the acidic Glu. In all cases, significantly higher CCS differences between the diastereomers were obtained with trimers bound to one copper ion than in previous reports in the literature.

There are several limitations of using this specific approach; namely, there are needs for: (i) specific chiral reference compounds and metal for each analyte, (ii) several experiments for optimization of the metal ion in chiral reference to analyte ratios, (iii) forming diastereomers in sufficiently high abundances; and (iv) minimization of ion suppression in electrospray ionization induced by metal ions. Moreover, there was no report of applying these approaches to mixtures of amino acids.

### 5.2. Tert-Butoxycarbonyl Modified Amino Acid as Chiral Selector

Due to the above-mentioned disadvantages, metal-free approaches were employed for chiral discrimination. Zhang et al. reported the use of DMS-MS in the chiral discrimination of Trp and Phe [107] and Cys and Pro [108]. In both cases, modified Ser with a tert-butoxycarbonyl group (namely, N-tert-butoxycarbonyl-O-benzyl-L-Ser -BBS) was used as chiral selector for forming diastereomeric complexes. Chiral separation of [(^DL^AA)(BBS)+H]^+^ diastereomers by DMS-MS achieved baseline separation of the spectral features as a function of the helium proportion in the He:N_2_ carrier gas mixture. Ions were more highly dispersed in the presence of a higher concentration of He (50%), resulting in better separations, except for proline diastereomers [(^DL^Pro)(BBS) + H]^+^, in which case a baseline separation was not achieved, but chiral discrimination of enantiomers was still possible.

### 5.3. Separation of Diastereomers after CDA Labeling

The use of a chiral derivatization agent in diastereomer formation was also reported. Pérez-Míguez et al. [81] used TIMS for the analysis of 17 amino acid enantiomers. Diastereomers were obtained by reaction with the chiral reagent (+)-FLEC, and were further separated by their mobility in a TIMS-TOF instrument. In the preliminary studies, two amino acids were used: DL-Orn and DL-SeMet. Only in the case of disodiated ions ([FLEC_x_ − AA + 2Na − H]^+^) did the TIMS analysis reveal two peaks separated at the baseline for each of the diastereomers. Instead, both protonated and sodium-containing ions ([FLEC_x_ − AA + H]^+^, [FLEC_x_ − AA + Na]^+^) corresponded to individual peaks for both of the AAs analyzed, thus no separation of diastereomers occurred. The method was used in the analysis of and D- and L-AA mixtures (1:1 and 1:3 ratios) after FLEC derivatization, confirming that separation by TIMS of the diastereomers was caused by structural differences. Additionally, in the TIMS analysis of the FLEC-derivatized D- and L-AA mixture at a 1:3 enantiomer ratio, a higher peak intensity was obtained for the L-form. This group also studied the effects of alkali cation (Li^+^, Na^+^, K^+^) addition on the TIMS separation of the enantiomers of the prototype amino acids used. As the resolving power in TIMS depends on the voltage range and ramp time, these two parameters were optimized to achieve the best separation of each pair of the 21 initial amino acid diastereomers. In addition, sensitivity was dependent on the number of ions trapped in the first TIMS cells, influenced by the time of ion accumulation. This last parameter was studied in the range of 10–100 ms: most of the (+)-FLEC-AA signal intensities increased until 50 ms, with Ser showing the maximum intensity at 25 ms, and His at 100 ms. The optimized TIMS method was applied successfully in the separation of diastereomers of 17 AAs with several observations: (i) for Glu, Pro, Thr, and Ala, no enantioresolution was obtained; (ii) for the neutral, nonpolar amino acids Val, Ile, and Leu, the diastereomer separation was observed for the monosodiated species ([FLEC_x_-AA + Na]^+^); (iii) largely, diastereomer separation was observed for the disodiated FLEC-AAs ([FLEC_x_-AA + 2Na-H]^+^); (iv) Tyr, along with AAs containing two amino groups, were derivatized with two FLEC molecules; and (v) no positive effect on the separation of the diastereomers was achieved with Li+ and K+.

(S)-naproxen chloride ((*S*)-NAP) chiral derivatization of amino acids and TIMS-MS analysis was employed by Will’s group [125]. Interestingly, the authors coupled an integrated chromatography system (ICS) to a TIMS-MS instrument. The ICS performed inline dilution of the sample, (*S*)-NAP derivatization, and pre-IMS separation of amino acid diastereomers using a strong cation exchange (SCX) column, with the eluted diastereomers being subjected to electrospray ionization and TIMS-MS. The AA diastereomers were successfully separated in negative mode as [(*S*)-NAP-DL-AA-H]^−^ or [(*S*)-NAP-DL-AA-H + NaOAc]^−^ in the case of Ser, Val, and Gln. S-NAP derivatization provided good resolution, especially for small AAs, allowing for the separation of eight amino acids (Ser, Ala, Met, Val, Gln, Phe, Tyr, and Trp).

In a recent study, Fukui et al. [102] compared three derivatization methods for discrimination of DL-2HG using cylindrical FAIMS. Consequently, three CDAs were used, namely DATAN, DMT(S)A, and the acetic anhydride of DMT(S)A. In terms of separation efficiency, only DMT(S)A derivatives of D- and L-2HG were separated in FAIMS, with a peak resolution of 0.9. The authors also tested a traveling-wave IMS in the differentiation of DMT(*S*)A-DL-2HG; however, no sufficient separation was obtained.

Ion-mobility mass spectrometry successfully joins size- and mass-selective separation in the analysis of biomolecules. The technique has clear analytical advantages in the separation of enantiomers in the form of an increased peak capacity, a reduction in chemical noise, and consequent augmentation of low-abundance analytical signals. Nevertheless, the separation of isomers in biological mixtures would require a higher IMS resolving power than the capabilities of current available instruments, assuring full analytical utility of IMS when coupled with MS and orthogonal chromatographic approaches. A combined data-acquisition and processing strategy for enhancing the resolving power of DT-IMS by multiplexing was reported in the separation of several isomeric mixtures [126,127,128]. This approach was reported recently by Demelenne et al. [128] for the separation of coeluting diastereomers of oligonucleotides containing different numbers of phosphorothioate (PS) linkages; the authors reported a 3.8-fold increase in the resolving power using this strategy. Moreover, adjacent diastereomers with differences in ^DT^CCS_N2_ values ranging from 0.9% to 2.9% were separated using this approach.

## 6. Conclusions and Perspectives

The developments in indirect chiral analysis methods over the recent years have been very significant in terms of developing new CDAs, analytical approaches to complex questions, and the development of new analytical technologies.

The most important aspect regarding the new developments in terms of CDAs probably has been the introduction of new CDAs to improve the sensitivity and specificity of MS detection; this was achieved by incorporating a permanent charge in their structure or by forming diastereomers that generate analyte specific fragments useful in MS/MS detection, respectively. Even though mass spectrometry is the most popular detection method nowadays in metabolomics studies, new CDAs have also been developed for optical detection, offering improved selectivity or faster derivatization (such as MAD). Nevertheless, considering that in many cases, the target of chiral metabolomics is to detect and quantify trace levels of metabolites, more emphasis should be put on determining the chiral purity of the CDAs in order to assure method accuracy.

Several relevant strategies for untargeted chiral metabolomics have been developed. Some aimed at observing D-AA ratios between control and diseased groups using isotope-labeled CDAs in order to screen for possible biomarkers; others focused on differentiating chiral molecules in an untargeted data set by employing both enantiomeric forms of a CDA. These innovative approaches have proved very useful already in identifying potential chiral biomarkers for different diseases.

A prerogative for generating relevant leaps in the knowledge of chiral metabolomics is the availability of high-throughput analysis methods. Still, there are not that many analytical methods capable of offering baseline separations for all proteinogenic amino acids in a reasonable amount of time (<20 min). A solution to this problem may be found in using complementary separation techniques, such as ion-mobility separations. Such approaches have been reported by several studies in the last few years, with notable results. Still, IMS-only separations of diastereomers is rather limited due to the low resolving power of today’s instruments. Nevertheless, combined with separative techniques, the measurement of CCS values can be useful in improving the annotation of chiral metabolites in complex samples.

## Supplementary Materials

The following supporting information can be downloaded at: https://www.mdpi.com/article/10.3390/ijms23137428/s1.
