# Supplementary material for: Indirect Enantioseparations: Recent Advances in Chiral Metabolomics for Biomedical Research
Source: Int J Mol Sci. 2022 Jul 4;23(13):7428. doi: 10.3390/ijms23137428 (PMC9267260; doi:10.3390/ijms23137428)
Supplement: Supplementary file 1 [file ijms-23-07428-s001.zip › ijms-1784286-supplementary.pdf]

Table S1: Indirect separation conditions reported in original research articles

Table S2: Strategies implemented in untargeted chiral metabolomics studies

Table S3: IMS of chiral AAs. Studies reporting technologies operating in low electric fields

Table S1. Indirect separation conditions reported in original research articles

Analytes	Matrix	CDA	Analytical technique	Separation conditions	Run time	LOD [M]	Ref.
**Amino acids**	Protein hydrolisate, standards	FDAA	LC-MS	Stationary phase: C18; Mobile phase: A:FA/NH_4Ac 5 mM, B: ACN+FA/ACN; Gradient elution	60 min	pM range	[82]
**Amino acids α-hydroxy acids**	Depsipeptide	FDAA	LC-MS	Stationary phase: C18; Mobile phase: A: FA, B: MeOH; Gradient elution	n.r.	n.r.	[43]
**Amino acids**	Tissue	FDLA	LC-MS	Stationary phase: C18; Mobile phase: A: HCO_2NH_4 5 mM, B: MeOH; Gradient elution	n.r.	n.r.	[46]
**All proteinogenic amino acids**	Standards	FDLA	LC-MS	Stationary phase: C18; Mobile phase: A: FA, B: ACN; Gradient elution	35 min	n.r.	[47]
**Amino acids**	Protein hydrolisate, standards	OPA/IBLC	LC-MS	Stationary phase: C18; Mobile phase: A:FA/NH_4Ac 5 mM, B: ACN+FA/ACN; Gradient elution	60	pM range	[82]
**Amino acids**	Serum, plasma, urine	OPA/IBLC	LC-HRMS	Stationary phase: C18; Mobile phase: A: NH_4Ac 2.5 mM B: ACN+MeOH	34	7.1-159.2 pM	[71]
**Trp**	Plasma, serum	OPA/NAC	LC-CL	Stationary phase: C18; Mobile phase: A: KH_2PO_4 (pH 6.0), B: MeOH; Isocratic elution	n.r.	<1.5 pM	[72]
**Primary and secondary amines**	Standards	OPA/NAC, FLEC	LC-FL	Stationary phase: C18; Mobile phase: A: NaAc 50 mM (pH 5.6), B: MeOH; Gradient elution	90 min	0.1-0.25 µM	[73]
**All proteinogenic amino acids**	aCSF	FLEC	MEKC-UV	BGE: 40 mM borate buffer (pH-9.2), 21 mM SDS, 8.5%IPA,	80 min	5 µM	[76]
**All proteinogenic amino acids**	aCSF	FLEC	MEKC-MS	BGE: 150 mM APFO (pH-9.5)	35 min	4-11 µM	[77]
**All proteinogenic amino acids**	CSF	FLEC	MEKC-MS	BGE: 150 mM APFO (pH-9.5)	50 min	0.4-5 µM	[78]
**Ser, Asp, Asn, Glu, Gln**	aCSF	FLEC	CE-MS	BGE: 150 mM acetic acid, (pH 3.7)	25 min	0.19-0.36 µM	[80]
**13 proteinogenic amino acids**	Standards	FLEC	MEKC-FL	BGE: 40 mM borate buffer (pH-9.2), 25 mM SDS, 15%IPA	40 min	13-580 nM	[79]
**All proteinogenic amino acids**	Standards	FLEC	LC-MS	Stationary phase: Diphenyl / Biphenyl, Mobile phase: A: FA 50 mM, B: ACN; Gradient elution	30 min	n.r.	[75]
**Amino acids**	Standards	FLEC	IM-MS	-/-	n.r.	n.r.	[81]

Analytes	Matrix	CDA	Analytical technique	Separation conditions	Run time	LOD [M]	Ref.
**All proteinogenic amino acids**	Plasma, urine, CSF	NIFE	LC-MS	Stationary phase: C18; Mobile phase: A: NH_4HCO_3 10 mM (pH 9.5), B: ACN; Gradient elution	21 min	<5 nM	[50]
**Amino acids**	Protein hydrolisate, standards	NIFE	LC-MS	Stationary phase: C18; Mobile phase: A:FA/NH_4Ac 5 mM, B: ACN+FA/ACN; Gradient elution	60 min	pM range	[82]
**Amino acids**	Plasma	NIFE	LC-MS	Stationary phase: C18; Mobile phase: A: NH_4HCO_3 8 mM, B: ACN; Gradient elution	18 min	0.4-341.4 pM	[85]
**Amino acids**	Brain homogenates	NIFE	LC-MS	Stationary phase: C18; Mobile phase: A: NH_4HCO_3 8 mM, B: ACN; Gradient elution	41 min	0.4-95 pM	[86]
**Epinephrine, norepinephrine**	Plasma	NIFE	LC-MS	Stationary phase: C18; Mobile phase: A: FA, B: ACN; Gradient elution	n.r.	5-8.86 pM	[84]
**Amino acids**	Milk	NIFE	LC-HRMS	Stationary phase: C18; Mobile phase: A: NH_4HCO_3 8 mM, B: ACN; Gradient elution	24 min	67 pM – 58 nM	[83]
**Amino acids**	Human nails	DBD-PyNCS	LC-HRMS	Stationary phase: C18; Mobile phase: A:FA/NH_4Ac 5 mM, B: ACN+FA/ACN; Gradient elution	35 min	0.5-375 nM	[110]
**Amino acids**	Plasma, nails	DBD-PyNCS	LC-HRMS	Stationary phase: C18; Mobile phase: A: TFA/FA, B: ACN/MeOH; Gradient elution	n.r.	n.r.	[31]
**Trp, KYN**	Serum	DBD-PyNCS	LC-MS	Stationary phase: HILIC; A: HCO_2NH_4 10 mM (pH 5.0), B: ACN; Isocratic elution	15 min	50 pM Trp, 4.0 pM KYN	[88]
**Ser**	Serum	DBD-PyNCS		Stationary phase: HILIC; Mobile phase: A: HCO_2NH_4 100 mM, B: ACN; Isocratic elution	n.r.	55 pM	[87]
**Trp, KYN**	Standards	DBD-PyNCS		Stationary phase: HILIC; Mobile phase: A: HCO_2NH_4 10 mM (pH 5.0), B: ACN; Isocratic elution	n.r.	5.1-19 nM	[89]
**Lactic acid**	Saliva (DSS)	NBD-Apy	LC-FL, LC-MS	Stationary phase: C18; Mobile phase: A: FA,ACN, B: MeOH, THF; Isocratic elution	20 min	FL: 33.3 nM MS: 6.6 nM	[24]
**Hydroxy/ amino acids**	Bone marrow, plasma	DATAN	LC-HRMS	Stationary phase: C18; Mobile phase: A: HCO_2NH_4 10 mM (pH 3.6), B: ACN/MeOH; Gradient elution	75 min	n.r.	[91]
**LA**	CSF, urine	DATAN	LC-MS	Stationary phase: C18; Mobile phase: A: HCO_2NH_4 2.5 mM (pH 3.6), B: ACN; Isocratic elution	n.r.	n.r.	[96]
**2HG**	Kidney tissue	DATAN	LC-MS	Stationary phase: C18; Mobile phase: A: HCO_2NH_4 (pH 3.6), B: ACN; Gradient elution	25 min	n.r.	[93]
**2HG**	Serum	DATAN	LC-MS	Stationary phase: C18; Mobile phase: A: HCO_2NH_4 2 mM (pH 3.1), B: ACN; Gradient elution	n.r.	60 nM	[53]
**2HG**	Serum, plasma, bone marrow, urine	DATAN	LC-MS	Stationary phase: C18; Mobile phase: A: HCO_2NH_4 (pH 3.6), B: ACN; Gradient elution	30 min	n.r.	[112]
**Amino and carboxyls compounds**	Serum, saliva, brain homogenates	DMT-Pro-OSu/ DMT-Apy	LC-MS	Stationary phase: C18; Mobile phase: A: FA, B: ACN; Gradient elution	60 min	n.r.	[100]

Analytes	Matrix	CDA	Analytical technique	Separation conditions	Run time	LOD [M]	Ref.
**Amino acids, amines**	Saliva	DMT-Pro-OSu	LC-MS	Stationary phase: C18, ADME; Mobile phase: A: different buffers, B: ACN, MeOH, THF; Isocratic elution	n.r.	Amines: 0.02-2.9 nM, AA: 0.04-3.2 nM, Ser: 542.6 pM	[55]
**Carboxylic acids**	CSF	DMT-A/iDMT-A	LC-MS	Stationary phase: C18; Mobile phase: A: FA, B: ACN; Gradient elution	60 min	0.04-44.25 nM	[101]
**Amines**	CSF	DMT-(S)-PO/iDMT-(S)-PO	LC-MS	Stationary phase: C18; Mobile phase: A: FA, B: ACN; Gradient elution	60 min	0.04-44.25 nM	[101]
**Carboxylic acids**	Standards, biological samples	DMT-Apy	LC-MS	Stationary phase: C18; Mobile phase: A: FA, B: ACN; Isocratic elution	n.r.	n.r.	[98]
**Amines, amino acids**	Standards, biological samples	DMT-Pro-OSu	LC-MS	Stationary phase: C18; ADME, Mobile phase: A: different buffers, B: ACN, MeOH, THF; Gradient elution	n.r.	n.r.	[98]
**Amino acids**	Crystallin	DMT-Pro-Osu	LC-MS	Stationary phase: C18; Mobile phase: A: FA, B: ACN; Gradient elution	120 min	n.r.	[99]
**LA**	Saliva	DMT-Apy	LC-MS	Stationary phase: C18; Mobile phase: A: FA, B: ACN; Isocratic elution	n.r.	6.00 pM	[22]
**Carboxylic acids**	Saliva	DMT-Apy	LC-MS	Stationary phase: C18; Mobile phase: A: FA, B: ACN; Gradient elution	20 min	3 pM - 330 nM	[54]
**2-HG**	Human serum	DMT-Apy	FAIMS/MS	-/-	-	-	[102]
**GSH, Cys, Hcy**	Saliva, urine	NCS-OTPP	LC-HRMS	Stationary phase: C18; Mobile phase: A: FA, B: ACN+MeOH; Isocratic elution	25 min	19.2-57.6 nM	[56]
**GSH, Cys, Hcy**	Serum	NCS-OTPP	LC-HRMS	Stationary phase: C18; Mobile phase: A: FA, B: ACN+MeOH; Isocratic elution	25 min	2.4-7.2 nM	[57]
**LA, HB**	Saliva	PMP	LC-MS	Stationary phase: C18; Mobile phase: A: FA, B: ACN+MeOH; Isocratic elution		LA: 27 pM, HB: 57 pM	[23]
**HB**	Tissues	PMP	LC-MS	Stationary phase: C18; Mobile phase: A: HCO_2NH_4 5 mM (pH 4.0), B: ACN+MeOH; Gradient elution	26 min	0.8-2.8 nM	[106]
**Amino acids**	Standards	N-(4,6-Dichloro-[1,3,5]-triazine-2-yl)-L-leucine (DCT)	LC-UV	Stationary phase: C18; Mobile phase: A: TFA, B: ACN; Gradient elution	45 min	0.9-1.1 nM	[97]
**Amines, carboxylic acids**	Saliva	(S)-PyT-N/(R)-PyT-C	LC-MS	Stationary phase: C18; Mobile phase: A: FA, B: ACN; Isocratic elution	16 min	Amine: 0.1 nM, CA: 0.1 nM	[103]
**Amino acids**	Standards	Nap-Btz	LC-UV	Stationary phase: C18; Mobile phase: A: TEAP 10 mM (pH 3.5), B: ACN; Gradient elution	35 min	5-60 nM	[68]
**RS-PEA**	Plasma	PGA	LC-MS	Stationary phase: C18; Mobile phase: A: FA, B: ACN; Isocratic elution	n.r.	0.5-2 nM	[59]
**Amino acids**	Serum, yogurt	PGA-Osu	LC-MS	Stationary phase: C18; Mobile phase: A: FA, B: ACN; Isocratic elution	n.r.	0.25-1.6 nM	[60]

Analytes	Matrix	CDA	Analytical technique	Separation conditions	Run time	LOD [M]	Ref.
**AA**	Rat plasma	COXA-OSu	LC-MS	Stationary phase: HILIC; Mobile phase: A: HCO2NH4 10 mM+FA 10 mM, B: ACN+MeOH; Gradient elution	60 min	2.8-103.6 nM	[58]
**AA**	Human plasma	OTPTHE	LC-UV	Stationary phase: C18; Mobile phase: A: FA, B: ACN; Gradient elution	30 min	2.5 μM	[62]
**Amino acids**	Urine	BiAC	LC-MS	Stationary phase: Phenyl; Mobile phase: A: FA/NH4Ac 10 mM, B: ACN; Gradient elution	20 min	n.r.	[105]
**Amino acids**	Urine, plasma	D-BPBr	LC-MS	Stationary phase: C18; Mobile phase: A: H_2O, B: ACN; Gradient elution	n.r.	0.25nM-12μM	[63]
**Amino acids**	Urine	D-BPCl	LC-MS	Stationary phase: C18; Mobile phase: A: H_2O, B: ACN; Gradient elution	24 min	50pM-12 mM	[64]
**SeMet**	Standards	MBIC	LC-UV	Stationary phase: C18; Mobile phase: A: TEAP (pH 4.5), B: ACN; Isocratic elution	n.r.	n.r.	[69]
**SeMet**	Standards	NEIC	LC-UV	Stationary phase: C18; Mobile phase: A: TEAP (pH 4.6), B: ACN; Isocratic elution	n.r.	n.r.	[69]
**Carboxylic acids**	Newborn dried blood spot, human saliva	ANA	LC-MS	Stationary phase: C18; Mobile phase: A: HCO_2NH_4 10 mM, B: MeOH; Isocratic elution	n.r.	n.r.	[70]
**Amine metabolites**	Urine	DIPP-Ala-NHS (16O2)/DIPP-L-Ala-NHS (18O2)	LC-MS	Stationary phase: C18; Mobile phase: A: NH4Ac 10 mM, B: ACN; Gradient elution	51 min	n.r.	[83]
**2HG**	Synovium, serum	TSPC	LC-MS	Stationary phase: C18; Mobile phase: A: FA, B: MeOH; Gradient elution	32 min	0.04-24 nM	[67]
**Carboxylic acids**	Saliva	PCP2	LC-MS	Stationary phase: C18; Mobile phase: A: HCO2NH4 20 mM, B: ACN; Gradient elution	13 min	16-260 pM	[104]
**2HG**	Standards, human urine, tissue	TSPC	LC-MS	Stationary phase: C18; Mobile phase: A: FA, B: ACN+MeOH; Gradient elution	n.r.	0.12-0.1 nM	[67]
**Ser**	Plasma	(R)-1-Boc-2-PCC	LC-MS	Stationary phase: C18; Mobile phase: A: TFA, B: MeOH; Gradient elution	25 min	0.19 mM	[65]
**Ser**	Plasma, CSF	(R)-1-Boc-2-PCC	LC-MS	Stationary phase: C18; Mobile phase: A: TFA, B: MeOH; Gradient elution	35 min	n.r.	[111]
**Amino acids**	Standards	Cu (II), Pro	TW IMS	-/-	-	n.r.	[122]
**Amino acids**	Standards	BBS	DMS-MS	-/-	-	n.r.	[107]
**Amino acids**	Standards	BBS	DMS-MS	-/-	-	n.r.	[108]

Table S2. Strategies implemented in untargeted chiral metabolomics studies

Strategy implemented	Chiral metabolites	CDA	LC separation	MS detection	Application	Ref
**Isotope labeling strategy**	Amine groups	L-PGA<br>L-PGA-d_5	C18 column 1.7 µm particle size, 2.1 mm x100 mm	- triple quadrupole mass spectrometer<br>- ESI-MS/MS in positive mode	PEA spiked in rat plasma	[59]
	DL-AAs	L-PGA-OSu<br>l-PGA[d_5]-OSu			Differential analysis of the DL-Ala values in two different yogurt products	[60]
	Carboxyl/amine groups	DMT-(S,R)-Pro-OSu<br>DMT3(S,R)-Apy<br><br>iDMT-(S,R)-Pro-OSu<br>iDMT3(S,R)-Apy		- Q-TOF mass spectrometer<br>- ESI-MS/MS in positive mode<br>*DDA MS/MS of precursor ions; threshold 20000 counts	Chiral metabolomics for carboxyls and amines in AD CSF and comparison with Non-AD patients	[101]
	DL-AAs<br>Small peptides	DIPP-Ala-NHS ($^{16}O_2$)<br>DIPP-Ala-NHS ($^{18}O_2$)	C18 column 5 µm particle size, 2.1 x150 mm	- Orbitrap mass spectrometer	Relative and absolute quantification of amino acids in human urine	[110]
**Use of opposite enantiomers of CDA for derivatization**	Amine groups	DMT-(S,R)-Pro-OSu	C18 column 1.7 µm particle size, 2.1 mm x100 mm	- triple quadrupole mass spectrometer<br>- ESI-MS/MS in positive mode	• Chiral metabolomics of carboxyls in saliva of diabetic patients and healthy persons<br>• Chiral metabolomics fingerprinting and chiral metabolomics extraction for carboxyls and amines in brain homogenate of Alzheimer's disease patients	[100]
	Carboxyl groups	DMT3(S,R)-Apy				
	Hydroxy/amine groups	(+)/(−) DATAN		- Orbitrap mass spectrometer	Chiral Metabolic Profiling of peripheric blood and bone marrow plasma of AML patients	[91]

Abbreviations: L-PGA: l-Pyroglutamic acid; PEA: 1-phenylethylamine; AA: amino acid; L-PGA-OSu: l-pyroglutamic acid succinimidyl ester; DMT-(S)-Pro-OSu: (S)-2,5-dioxopyrrolidin-1-yl-1-(4,6-dimethoxy1,3,5-triazin-2-yl) pyrrolidine-2-carboxylate; DMT-3(S)-Apy: (S)-1-(4,6-Dimethoxy-1,3,5-triazin-2-yl)pyrrolidin-3-amine; DDA: data dependent analysis; AD: Alzheimer disease; CSF: cerebrospinal fluid; DATAN: diacetyl-tartaric anhydride; AML: acute myeloid leukemia

Table S3. IMS of chiral AAs. Studies reporting technologies operating in low electric fields

IMS type	Analysis form	Chiral reference compound	Analyte	m/z	D-AA CCS value (Å^2)	L-AA CCS value (Å^2)	ΔCCS	Δt$_d$	Rp-p	Ref.
TWIM-MS	[(**^{D}AAref)$_2$**+$^{D/L}$ Analyte+Cu^{II}–H]$^+$	D-Pro	Phe	457.14	123.5	127.3	3.8	0.11	0.44	[122]
			Arg	466.17	126.6	127.9	1.3	0.04	-	
			Glu	439.11	121.7	122.7	1.0	0.03	0.12	
			Thr	411.12	115.3	116.3	1.0	0.03	-	
			Gln	438.12	121.7	123.0	1.3	0.04	-	
			Lys	438.16	123.0	124.3	1.3	0.04	-	
			Tyr	473.13	126.3	130.7	4.4	0.14	0.50	
			Trp	496.15	127.5	133.7	6.2	0.18	0.60	
		D-Arg	Phe	575.23	119.7	121.1	1.4	0.06	-	
		D-Trp		635.19	155.8	157.0	1.2	0.04	-	
		D-Glu		521.12	115.4	115.7	0.3	0.05	-	
		D-Thr		465.13	146.4	147.7	1.3	0.04	-	
		D-His		537.15	147.5	148.8	1.3	0.04	-	
		D-Pro		457.14	123.5	127.3	3.8	0.01	-	
		D-Tyr		589.16	152.8	154.6	1.8	0.06	-	
	[(^{D}AAref)+$^{D/L}$Analyte+Cu^{II}–H]$^+$	D-Pro	Phe	342.07	102.5	103.9	1.4	0.04	-	
		D-Arg		401.12	119.7	121.1	1.4	0.04	-	
		D-Trp		431.10	122.4	123.0	0.6	0.02	-	
		D-Glu		374.06	115.4	115.7	0.3	0.01	-	
		D-Thr		346.07	104.3	105.0	0.7	0.02	-	
		D-His		382.08	110.5	111.2	0.7	0.02	-	
		D-Tyr		408.08	117.0	117.3	0.3	0.01	-	
	[(Cu^{II})$_{2+}$$^{D/L}$Analyte+(^{L}AAref)$_3$-4H+ H/Na]$^+$	His	Trp	814	176.4	164.3	12.1	0.65	1.8	[124]
			Gln	756	164.7	155.5	9.2	0.48	1.4	
			Tyr	791	171.9	161.7	10.2	0.54	1.4	
			Thr	729	160.1	156.0	4.1	0.21	0.7	
		Phe	His	795	171.1	175.9	4.7	0.26	0.7	
		Trp	Gln	903	191.3	180.8	10.5	0.59	1.5	
			Tyr	938	193.8	183.9	9.8	0.56	1.2	
			Glu	904	190.0	181.5	8.5	0.48	1.2	
			Met	906	193.4	187.0	6.4	0.37	0.9	
			Phe	922	194.7	189.5	5.2	0.30	0.7	
		Tyr	Glu	654	144.5	150.3	5.8	0.29	1.0	
			Arg	840	177.3	184.0	6.7	0.37	1.0	
			His	843	180.5	185.0	4.5	0.25	0.7	
TIMS	[FLEC-AA+Na–H]$^+$	-	Ile	390.1656	190.0	187.1	2.9	-	-	[81]
			Leu	390.1681	191.9	188.7	3.2	-	-	

			Val*	376.1526	1: 182.3; 2: 185.3		3.0	-	-	
	[FLEC-AA+2Na−H]$^+$		Asn	413.1083	194.4	183.9	10.5	-	-	
			Glu*	428.1080	1: 190.0; 2: 197.4		7.4	-	-	
			SeMet	478.0475	195.1	198.6	3.5	-	-	
			Aad	442.1193	195.4	199.0	3.6	-	-	
			Pipe	410.1342	187.9	192.4	4.5	-	-	
			Gln	427.1243	197.1	192.0	5.1	-	-	
			Pro	396.1175	1: 189.8		-	-	-	
			Thr	400.1117	1: 187.0		-	-	-	
			Arg	455.1700	199.7	202.5	2.8	-	-	
			Ala	370.1008	1: 180.4		-	-	-	
			Met	430.1046	189.8	192.2	2.4	-	-	
			Phe	446.133	196.8	190.6	6.2	-	-	
			Ser	386.099	185.1	181.0	4.1	-	-	
			Trp	485.1444	207.1	201.3	5.8	-	-	
	[FLEC$_2$-AA+2Na−H]$^+$		Orn	649.2304	239.4	242.9	3.5	-	-	
			Lys	663.2447	237.5	240.0	2.5	-	-	
			Tyr	698.2138	254.5	249.6	4.9	-	-	
			Hys	672.2054	245.5	248.3	2.8	-	-	
	[FLECx-AA+2Li]$^{2+}$		Orn*	617.2803	DL: 240.3		-	-	-	
			SeMet*	446.1019	DL: 193.3		-	-	-	
	[FLECx-AA+2Na]$^{2+}$		Orn*	649.2281	1: 237.7; 2: 241.5		3.8	-	-	
			SeMet*	478.0497	1: 194.9; 2: 198.3		3.4	-	-	
	[FLECx-AA+2K]$^{2+}$		Orn*	681.1775	1: 239.9; 2: 242.4		2.5	-	-	
			SeMet*	510.0001	DL: 203.8		-	-	-	
	[S-NAP-AA-H]$^-$	-	Ala	300.1240					1.0	[125]
			Asp	344.1140					0	
			Glu	358.1304					0	
			Met	360.1274					0.8	
			His	366.1468					0.4	
			Phe	376.1553					0.9	
			Tyr	392.1503					0.7	
			Trp	415.1663					0.7	
	[S-NAP-AA-H+NaOAc]$^-$	-	Ser	398.1227					1.1	
			Val	410.1582					1.9	
			Gln	439.1486					0.6	
